# The Human Dorsolateral Prefrontal Cortex in Pain and Pain Modulation: A Review and Activation‐Likelihood Estimation Approach

**DOI:** 10.1002/ejp.70318

**Published:** 2026-06-24

**Authors:** Lewis S. Crawford, Damien C. Boorman, James W. M. Kang, Allan Peng, Kevin A. Keay, Luke A. Henderson

**Affiliations:** ^1^ School of Medical Sciences (Neuroscience), the Brain and Mind Centre University of Sydney Camperdown Australia; ^2^ Department of Psychology University of Toronto Mississauga Mississauga Ontario Canada

**Keywords:** dorsolateral prefrontal cortex, endogenous analgesia, magnetic resonance imaging, pain, placebo analgesia

## Abstract

**Background:**

The dorsolateral prefrontal cortex (dlPFC) has become a cornerstone of pain neuroscience, believed critically involved in processing acute and chronic pain, as well as in pain modulation. The dlPFC is thought to balance sensory perception with the cognitive appraisal of noxious stimuli. In humans however, the dlPFC spans several cortical nodes, and little attention has been given to precisely which subareas are tethered to these delineable processes. The primary goal of this review and co‐ordinate based meta‐analysis was to precisely map dlPFC areas engaged under varied effects on overall pain perception.

**Methods:**

Here, we combined a stringent meta‐analytic approach of neuroimaging studies reporting dlPFC activation as a core finding (i.e., within title, abstract, or keywords of each manuscript), with activation likelihood estimation to determine dlPFC engagement across experimental acute pain (combined *n* = 814), chronic pain (combined *n* = 2467), and the pain modulatory phenomenon of placebo analgesia (combined *n* = 791), conditioned pain modulation (combined *n* = 184), and offset analgesia (combined *n* = 47). Through activation likelihood estimate and foci analysis, we produced schema of the similar and disparate locations of dlPFC activity within and between pain perceptual and modulatory effects.

**Results:**

During both acute and chronic pain and in placebo analgesia, we identified remarkably similar activation patterns predominantly within Brodmann areas 8, 9, and 46—with no clear laterality effect and the left and right cortical hemispheres engaged regardless of the site of experimentally applied or conditionally‐present pain. Offset analgesia and conditioned pain modulation appeared to preferentially engage Brodmann areas 8 and 45, and 9 and 46, respectively.

**Conclusions:**

Despite the dlPFC large cortical surface area, these findings suggest that both the percept and inhibition of pain by placebo engage a similar focussed area of this region. Better understanding of exactly which cortical components of the extensive cortical pain system are involved in pain perception and modulation is critical for refining novel treatments such as non‐invasive brain stimulation which seek to target and modulate specific sites of cortical processing to alleviate pain.

**Significance Statement:**

This meta‐analysis precisely maps the dorsolateral prefrontal cortex (dlPFC) subregions engaged across acute pain, chronic pain and major endogenous pain‐modulation paradigms. By integrating data from more than 4000 participants, it provides the most anatomically resolved evidence to date that dlPFC engagement is a shared feature of pain perception and modulation. These findings refine the cortical targets most relevant for therapeutic neuromodulation and strengthen the rationale for dlPFC‐focused interventions in pain treatment.

## 1 Introduction

Following the identification of the dorsolateral prefrontal cortex's (dlPFC) role in cognition over 50 years ago, it has since been established that this region is integrated into a wider network responsive to tasks that demand cognitive load, that is, an Executive Control Network (ECN) (Fox et al. 2005; Goldman and Rosvold 1970). Numerous functional magnetic resonance imaging (fMRI) studies have also begun to define the role of the dlPFC and ECN in the cognitive and affective aspects on pain, with multiple studies reporting altered dlPFC activation during acute pain, chronic pain and pain modulation (Bär et al. 2007; Bornhövd et al. 2002; Freund et al. 2007; Gracely et al. 2004; Grachev et al. 2003; Lorenz et al. 2003; MacDonald 3rd et al. 2000; Mongini et al. 2005; Oosterman et al. 2006; Seminowicz and Davis 2006; Seminowicz et al. 2004; Zubieta et al. 2005). However, despite the seemingly consistent activation patterns during pain perception and modulatory domains, the dlPFC is not one homogenous structure, but comprises multiple parcellations (Laurence 2005). Indeed, the recent human connectome project extended atlas describes the dlPFC as 22 independent parcellations per hemisphere, with their own unique cortical architecture, resting‐state connectivity, task‐based activation and topographic organization (Huang et al. 2022).

With this complex dlPFC architecture now better characterised, the role of precise dlPFC regions in acute and chronic pain remains unknown. Additionally, whether the same dlPFC regions display altered activity changes during both pain perception and pain modulation tasks such as placebo analgesia, conditioned pain modulation and offset analgesia, remains unresolved. A better understanding of the precise regions involved in various pain‐related functions is essential if we are to develop more targeted neuromodulatory techniques for pain management.

In this study we combined a PRISMA‐based meta‐analytic framework with activation‐likelihood estimation (ALE) performed in GingerALE software to determine where activation maxima during pain perceptual and modulatory paradigms within the 22 dlPFC subregions (Eickhoff et al. 2009). We first conducted a comprehensive search of the literature in PubMed, the Web of Science and Scopus. Records were screened and quality for inclusion was determined prior to extracting and tabulating peak activation co‐ordinates for the dlPFC for acute pain, chronic pain, or across our pain modulatory paradigms. ALE contrast maps were then generated and activation probabilities in each dlPFC subregion were compared across the left and right cortical hemisphere. We hypothesized that the majority of included literature would fall within the categories of acute pain and placebo analgesia, given the importance placed on the dlPFC specifically in this pain modulatory phenomenon, and that these activations across each domain of pain would overlap within discrete components of the dlPFC rather than equally activating its entire extent.

## 2 Methods

**FIGURE 1:**
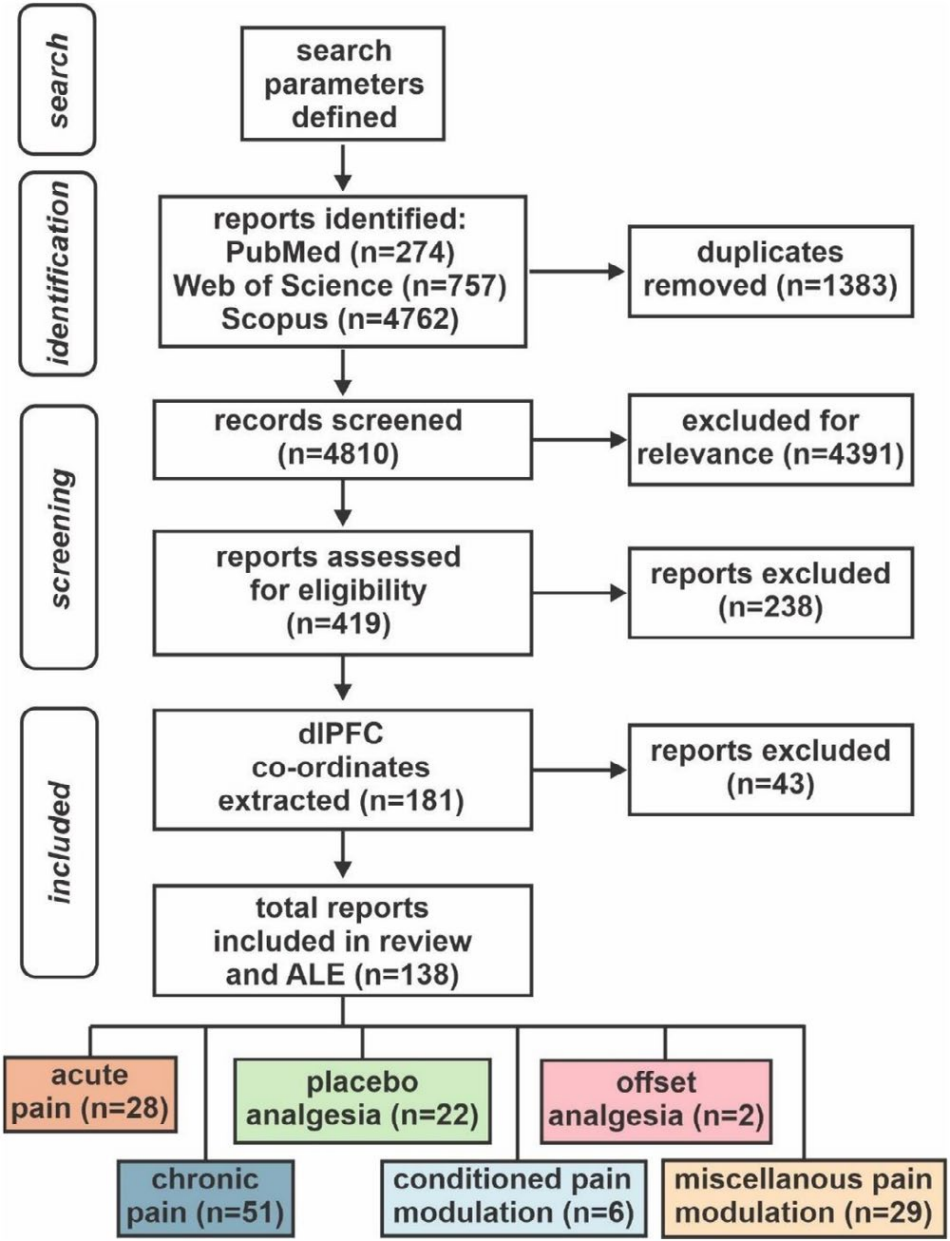
Flow chart for selection, screening and inclusion of records for meta‐analysis. A total of 138 studies were selected for inclusion across acute and chronic pain, as well as well‐known endogenous modulatory phenomena. Returned records are represented by ‘*n*‐values’ for identification, based on an initial search conducted on April 18th, 2024, with screening commencing on May 5th, 2024. Additional screening occurred at sixth month intervals preceding publication, that is, in November 2024 and May 2025. Records were initially deduplicated, before being manually screened for topic relevance. An additional screening period was conducted by two independent assessors where review articles and modalities unrelated to functional activation or connectivity change were excluded (*n* = 238). Resulting records were subject to template co‐ordinate extraction of cluster maxima, where a final screening was conducted where removal occurred if no co‐ordinate structure could be identified (*n* = 43). These records were split into six categories—acute and chronic pain, placebo analgesia, conditioned pain modulation (CPM), offset analgesia and miscellaneous pain modulation.

The initial search and screening was completed on the 5th of May, 2024 across the three databases: Pubmed, the Web of Science and Scopus (Table 1, Figure 1). Additional searches and screenings were conducted at six‐month intervals, with the final search being conducted on the 5th of May, 2025. The protocol was pre‐registered on the Open Science Framework (https://osf.io/tk89x).

**TABLE 1:** Meta‐analysis search terms and databases.

Database	Search terms	No. of reports returned
Pubmed	(Pain OR Chronic Pain OR Chronic Neuropathic Pain OR Pain Modulation OR Placebo OR Conditioned Pain Modulation OR Offset Analgesia OR Analgesia) AND (Dorsolateral Prefrontal Cortex OR dlpfc OR dlPFC) AND (Neuroimaging OR Magnetic Resonance Imaging OR MRI OR functional connectivity OR resting state OR rs‐fMRI)	611
Web of Science	(Pain OR Chronic Pain OR Chronic Neuropathic Pain OR Pain Modulation OR Placebo OR Conditioned Pain Modulation OR Offset Analgesia OR Analgesia) AND (Dorsolateral Prefrontal Cortex OR dlpfc OR dlPFC) AND (Neuroimaging OR Magnetic Resonance Imaging OR MRI OR functional connectivity OR resting state OR rs‐fMRI)	677
Scopus	(Pain OR Chronic Pain OR Chronic Neuropathic Pain OR Pain Modulation OR Placebo OR Conditioned Pain Modulation OR Offset Analgesia OR Analgesia) AND (Dorsolateral Prefrontal Cortex) AND (Neuroimaging OR Magnetic Resonance Imaging OR MRI OR functional connectivity OR resting state OR rs‐fMRI)	4762

*Note:* Reports from all publication dates were included, and no other filters or restrictions were instituted beyond the listed search terms. Database outputs were exported as .ris files and imported into Zotero for deduplication.

### 2.1 Dorsolateral Prefrontal Cortex Parcellation

**FIGURE 2:**
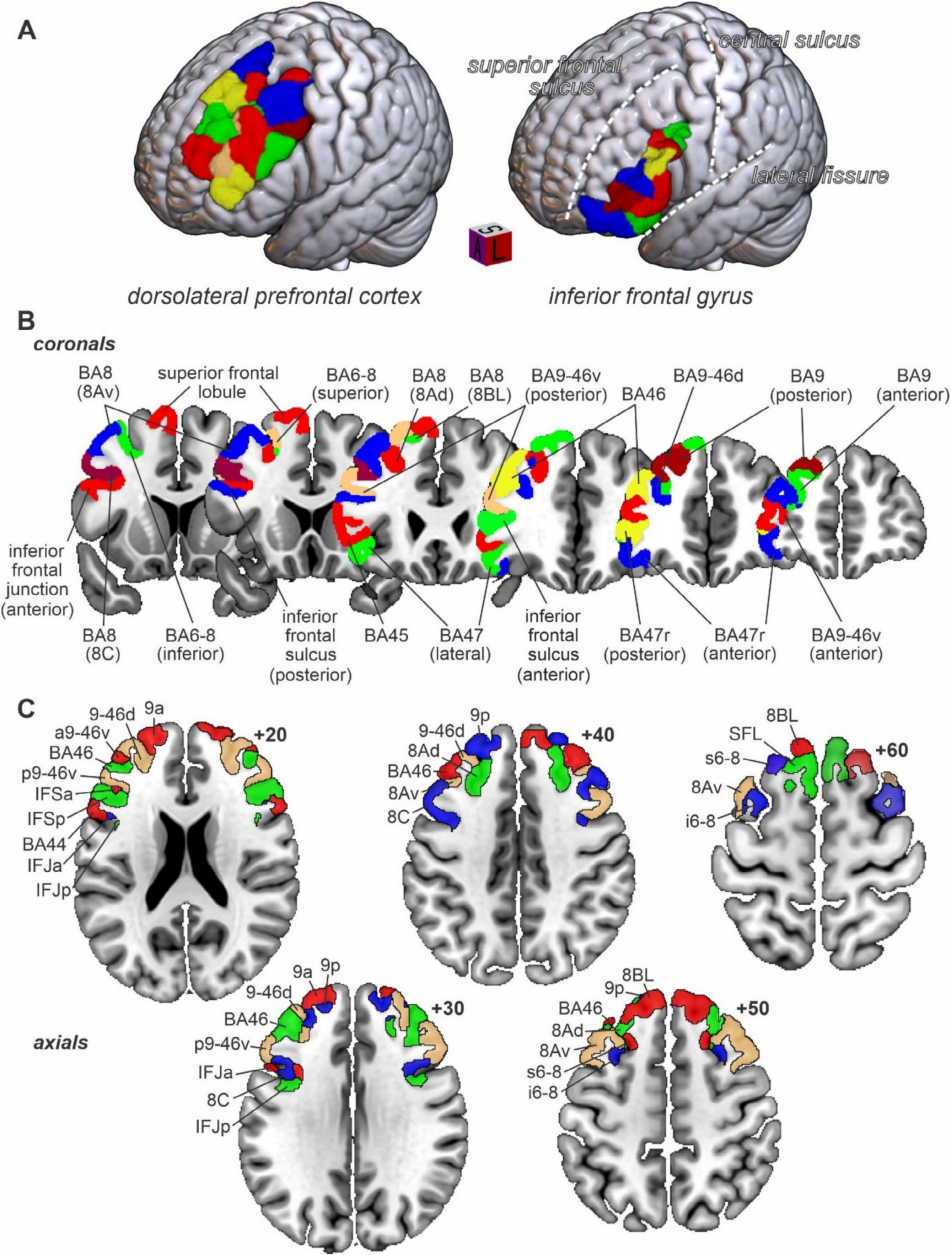
Anatomical Schematic of the Dorsolateral Prefrontal Cortex. The current prevailing human brain atlas—the human connectome project version 1.1 extended—defines the dorsolateral prefrontal cortex (dlPFC) as 13 independent parcels of grey matter per hemisphere, largely occupying Brodmann areas 8, 9 and 46. Often activation maxima residing within the inferior frontal gyrus are labelled as dlPFC in functional human brain imaging literature, which comprises an additional nine parcels per hemisphere, consisting of Brodmann areas 44, 45 and 47. This atlas was structured through inspection of cortical architecture, resting connectivity between parcels, task‐based activation and topographic organization in 210 participants. Although granular, this schematic provides a foundation to attempt to localize precisely which dlPFC subregions are involved in the multifaceted perception of pain and its endogenous modulation. Panel (A) Provides a rendered view of the dorsolateral prefrontal cortex, alongside anatomical landmarks which border it's position in the frontal lobe. Panels (B) and (C) delineate each parcel composing the dorsolateral prefrontal cortex in the left and right cortical hemispheres, in either coronal (B), or axial (C) orientation.

Using the human connectome project (HCP) version 1.1 extended, we used the dlPFC divisions which comprise 13 independent parcels of grey matter per hemisphere, largely occupying Brodmann areas 8, 9 and 46. Because fMRI activation maxima within the inferior frontal gyrus are often identified as dlPFC, we also included this region, which comprises an additional nine parcels per hemisphere, consisting of Brodmann areas 44, 45 and 47. See Figure 2 for schematic showing individual parcellations and Table 2 for Brodmann's areas and HCP atlas parcellation list.

**TABLE 2:** Dorsolateral Prefrontal Cortex (dlPFC) Parcels in each subregion of interest.

Brodmann area	HCP‐ex parcel list
BA 8	I6‐8, s6‐8, 8AD, 8Av, 8BL, 8C, SFL
BA 9	9a, 9p, 9‐46d, IFJa, IFJp
BA 44	44
BA 45	45
BA 46	A9‐46v, p9‐46v, 46, IFSa, IFSp
BA 47	47 l, a47r, p47r

*Note:* The original Brodmann 52‐region atlas was largely derived from human cytoarchitecture and cortical topology and remains today one of the most influential bodies of work in human neuroscience. This atlas is the foundation for most modern parcellations of the human cortex, of which the Human Connectome Project Extended (HCP‐ex) is no exception. The HCP‐ex iterated on the Brodmann atlas by including features of resting and task‐based functional connectivity, as well as cytoarchitecture and cortical topography; however, these distinct parcels can be aligned with their original Brodmann‐52 counterparts, with increased granularity.

Abbreviations: 44, brodmann area 44; 45, brodmann area 45; 46, brodmann area 46; 47 L, lateral brodmann area 47; 8Ad, anterodorsal brodmann area 8; 8Av, anteroventral brodmann area 8; 8BL, basolateral brodmann area 8; 8C, central brodmann area 8; 9‐46d, dorsal intermediate area 9 to 46; 9a, anterior brodmann area 9; 9p, posterior brodmann area 9; a47r, anterorostral brodmann area 47; A9‐46v, anterioventral intermediate area 9 to 46; I6‐8, inferior intermediate area 6 to 8; IFJa, anterior inferior frontal junction; IFJp, posterior inferior frontal junction; IFSa, anterior inferior frontal sulcus; IFSp, posterior inferior frontal sulcus; p47r, posterorostral brodmann area 47; p9‐46v, posteroventral intermediate area 9 to 46; S6‐8, superior intermediate area 6 to 8; SFL, superior frontal lobule.

### 2.2 Search Strategy

To provide an accurate appraisal of the existing literature for this meta‐analysis, we employed a scoping review approach aligning with the Preferred Reporting Items for Systematic Reviews and Meta Analysis (PRISMA) guidelines (Moher et al. 2009). Minor changes were made from the preregistered protocol in selecting the final method of report screening, based on the initial output of preliminary scoping procedures. Specifically, due to the conceptual disparity between experimentally induced pain and persistent chronic pain subtypes, independent search terms were entered to encompass both pain presentations. The search terms were designed to capture human brain imaging research output focusing on (1) an acute or chronic pain state; (2) endogenous pain modulatory phenomena which were evoked to either of these states; and (3) dlPFC function relating to either (1) or (2). Resulting record lists were then imported into the reference management software (Zotero, version 6), and manually deduplicated. The search terms used are presented in Table 1.

### 2.3 Selection of Studies

Records were filtered and assessed for inclusion based on the Condition, Context and Population (CoCoPop) framework. The Conditions of interest were acute pain, chronic pain and 3 pain modulatory phenomena: placebo analgesia, conditioned pain modulation and offset analgesia. The Context was functional human brain imaging, either task‐based or resting state connectivity. The Populations required at least one participant group—either pain‐free controls or a group with a chronic pain condition, with some imaging change being identified within the dlPFC as relating to these conditions. Blinded screening of abstracts was performed by two authors (LC, AP), with resulting lists compared for consistency. Discrepancies were resolved by decisions made from the corresponding author (LAH). To be eligible for inclusion, records were required to: (1) be published in English; (2) be published between the years 1999–2025; (3) be a primary research article; (4) involve whole brain analyses; and (5) report activation maxima for imaging changes in the dlPFC. Published pilot data and conference abstracts not published as full manuscripts were excluded. Further, records were excluded if pre‐determined region‐of‐interest masks were used for data extraction, or no co‐ordinate maxima within standard stereotaxic space (either MNI or Talairach) could be identified within the manuscript.

### 2.4 Data Extraction

For all included studies, we extracted data relating to group sizes, year of publication, body side (left or right) of either experimentally applied or present pain, and the type of image analysis performed leading to neural changes within the dlPFC. Directionality (i.e., increases or decreases) of signal relating to each condition or modulatory phenomenon was also extracted. Following data extraction, a structured risk‐of‐bias summary of included studies was performed following a modified version of the Risk of Bias in Non‐randomized Studies—of Interventions (ROBINS‐I) framework. Sources of bias emerging from: study design; randomization procedures; blinding of outcome assessment; reporting on withdrawal and drop out; listed inclusion or exclusion criteria; and reporting on adverse events were assessed for likely low, moderate and high risk of bias. Additionally, for construction of Activation Likelihood Estimation contrast images, stereotaxic co‐ordinate maxima were extracted from each instance of dlPFC activity within presented tables of records. That is, if both the left and right dlPFC were identified as being altered by the study's experimental methods, then both these co‐ordinates were extracted and used in ALE analyses. Whilst initially we screened for studies concerning the pain modulatory phenomena placebo analgesia, conditioned pain modulation and offset analgesia, records were identified in acute and chronic pain where some other modulatory mechanism was being investigated. To further resolve dlPFC function across pain modulatory effects, data were extracted from these records and collated as a category ‘miscellaneous pain modulation’. These records encompassed non‐invasive brain stimulation; expectancy and emotional pain modulation; attentional analgesia; musical analgesia; hypnosis; and exercise‐induced analgesia.

### 2.5 Activation Likelihood Estimate Analyses

In order to generate a summary of the dlPFC changes relating to each pain presentation and modulatory phenomenon, BrainMap GingerALE 3.0.2 was used to create six separate activation likelihood estimation (ALE) contrasts from each of the MNI co‐ordinates reported across included records on pain perception (acute; chronic) and modulation (placebo analgesia, conditioned pain modulation, offset analgesia, miscellaneous pain modulatory mechanisms) (Eickhoff et al. 2009). Articles reporting cluster maxima in Talairach space were converted to MNI using SPM conversion within the GingerALE software. For each of the six effects of interest, single study analysis was performed at an initial uncorrected voxel‐wise threshold of *p* < 0.001 uncorrected, with cluster level correction applied at a family‐wise error level of *p* < 0.05 with 1000 threshold permutations. ALE co‐ordinate data was then displayed on the MNI152 normalized template within the image viewing software MRICROGL version 1.2.20220720 (Rorden 2025). All 22 dlPFC parcels in each cortical hemisphere of the human connectome project extended atlas were then overlayed to the MNI152 template alongside each of the ALE maps to delineate within which area of the dlPFC significant clusters were identified. Under the ALE framework, resulting clusters represent groups of voxels with similar spatial convergence across included records' reported maxima of dlPFC activation. Surviving clusters with a volume greater than 32 mm and the individual parcels they occupied were extracted and tabulated between pain and pain modulatory conditions of interest, for qualitative analysis of where across included records significant dlPFC activity clustered within distinct Brodmann areas.

### 2.6 Dorsolateral Prefrontal Cortex Frequency Analysis by Condition

To delineate more specifically the proportion of records identifying maximal dlPFC neural changes in each dlPFC parcel between pain and pain modulatory conditions, the maximal MNI co‐ordinate of each record was entered into MRICROGL, with the normalized MNI152 template and HCP extended atlas overlayed. For each record, the dlPFC parcel identified as significantly altered was recorded, and a frequency analysis conducted across each of the six conditions. Dissimilar to the ALE, frequency testing of precise record foci reveals how commonly discrete dlPFC parcels show maximal engagement across pain perception and modulation, rather than commonly converging voxels in a cluster mass. That is, a percentage of the total foci in each condition was calculated for each Brodmann area of the left and right cortical hemispheres to determine whether certain conditions showed laterality in their engagement of the dlPFC, or whether between conditions, specific areas of the dlPFC were being preferentially engaged in a similar manner.

## 3 Results

A total of 6193 records were returned upon initial screening. Once imported to Zotero, 1383 duplicates were removed resulting in 4810 records being screened for eligibility. 4391 records were excluded as irrelevant to the topic area or not satisfying inclusion criteria, leading to 419 records being assessed further for eligibility. Of these records, 238 were deemed ineligible based on not recording functional imaging data, and a further 43 were removed as they did not report dlPFC co‐ordinates. The final 138 records were then delineated into pain and pain modulatory conditions of interest, with a total of 28 records allocated to *acute pain states*, 51 to *chronic pain states*, 22 to *placebo analgesia*, 6 to *conditioned pain modulation*, 2 to *offset analgesia* and 29 to *miscellaneous pain modulation* (Figure 1). 2 studies from the acute pain, 27 from the chronic pain, and 7 from the four pain modulation conditions were resting functional connectivity studies; the remaining studies involved evoked signal intensity changes. Table S1 provides a list of all records included in each of these six categories. Most included studies returned low or moderate potential risk of bias to each of the six categories of our modified ROBINS‐I framework. Notable exceptions occurred in two domains, and predominantly within the records encompassing *chronic pain* states. Specifically, potential high risk of biases was identified in randomization procedures and in the reporting of adverse events. Since the focus of this meta‐analysis was to determine common or disparate activation patterns within the dlPFC related to pain states and pain modulatory paradigms, and given no included records indicated above a moderate risk of bias in experimental design, no records were removed as a result of this risk of bias assessment. Table S2 provides a structured risk‐of‐bias summary table of all included records.

### 3.1 Directionality, Lateralization and Engagement of the dlPFC During Acute Pain and Pain‐Free and Chronic Pain States

**FIGURE 3:**
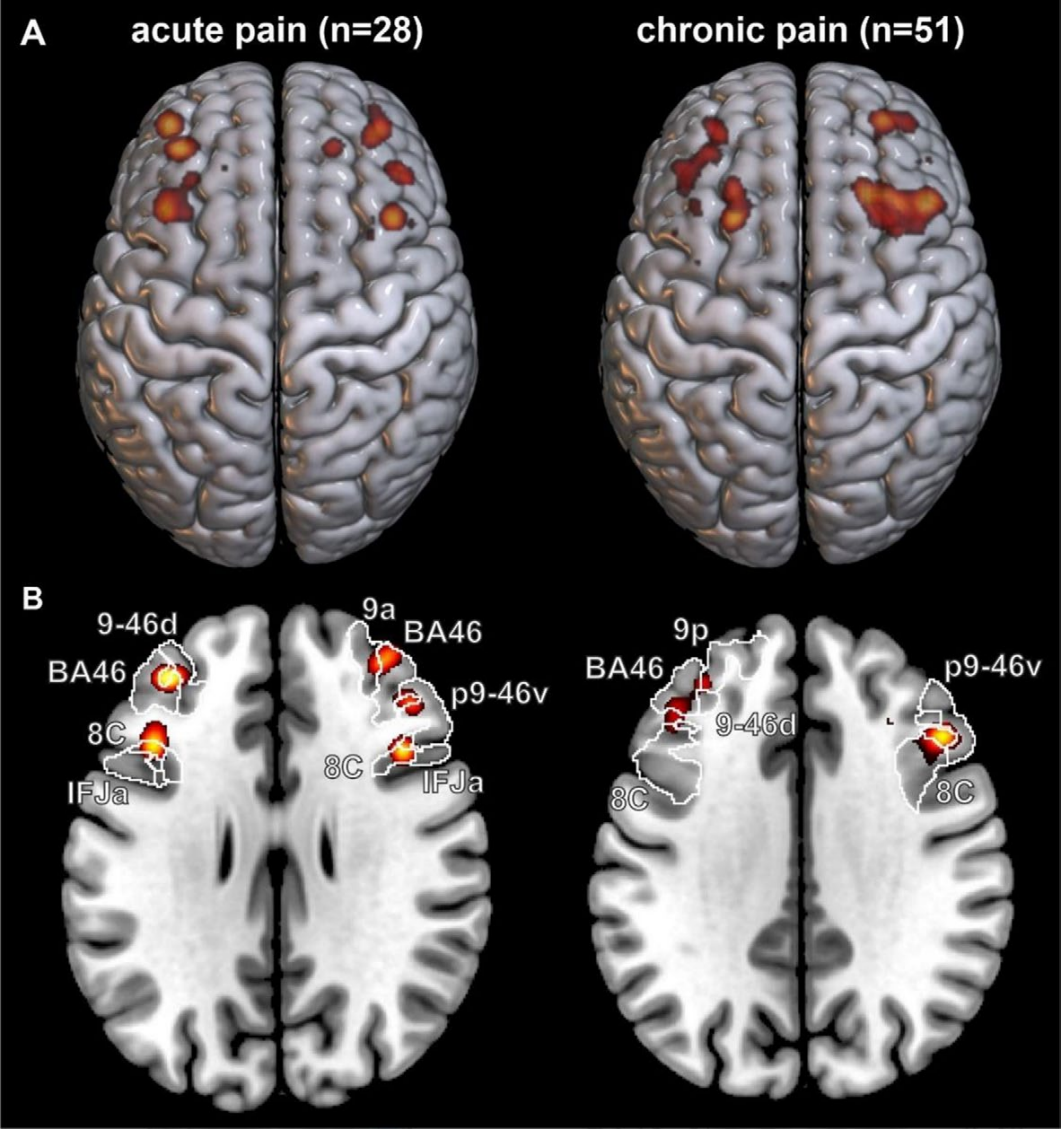
Activation Likelihood Estimate (ALE) pattern masks of dorsolateral prefrontal involvement in acute and chronic pain. (A) Forty‐five and seventy‐five foci were identified across screened records of dlPFC engagement in acute (28 records) and chronic (51 records) pain, respectively. Despite flipping maximally activated co‐ordinates on the midline to simulate as if all noxious stimuli had been applied to the left body side, both acute and chronic pain dlPFC activation appear to emerge bilaterally across experimental investigations. (B) Of these clustering, the predominantly activated regions appear in sub‐parcels of brodmann areas 46, 8 and 9. Note that these ALE maps were generated at an uncorrected *p*‐value threshold of *p* < 0.001, with a minimum cluster forming volume of 32 mm within Ginger‐ALE software version 3.0.2.

The location of dlPFC regions displaying significant signal intensity changes during acute pain stimuli in pain‐free controls and in individuals with chronic pain is shown in Figure 3. Noxious stimuli evoked signal changes in similar dlPFC regions in both groups. During acute pain stimulation in pain‐free controls, signal changes were bilateral, almost exclusively increases in nature (25 of 28 studies: 89%) and were located most frequently in the areas encompassing the left and right 8C, 46 and 9‐46d. Studies that reported signal decreases were most frequently located in areas 8Av and 9‐46d. Resulting ALE clusters of significance were also identified bilaterally, with five isolated to the left and six to the right cortical hemisphere. As in the analysis of individual peaks, ALE clusters predominantly occupied BA 8, 9 and 46 (Table 3).

**TABLE 3:** Activation likelihood estimate clusters within each pain and pain modulatory condition surviving volume thresholding at 32 cubic millimetres.

Condition	Cluster number	Cluster volume (mm)	Peak cluster MNI co‐ordinate [x y z]			dlPFC occupying parcels	Brodmann area
Acute pain	1	944	−38	50	15	L_a9‐46v*; L_9‐46d	L_BA46*; L_BA9
	2	950	−36	42	28	L_BA46*; L_9‐46d	L_BA46*; L_BA9
	3	979	40	18	30	R_8C*; R_IFJa; R_IFSp	R_BA8*; R_BA9; R_BA46
	4	2024	−40	20	30	L_8C*; L_IFJa; L_IFSp; L_8Av	L_BA8*; L_BA9; L_BA46
	5	1237	34	48	28	R_BA46*; R_9‐46d	R_BA46*; R_BA9
	6	1184	44	34	26	R_p9‐46v*; R_IFSp	R_BA46*
	7	422	19	42	42	R_8Ad*; R_8BL	R_BA8*
	8	107	48	16	50	R_8Av*	R_BA8*
	9	106	33	12	48	R_I6‐8*	R_BA8*
	10	89	−44	8	44	L_8C*; L_8Av	L_BA8*
	11	52	−20	34	46	L_8Ad*	L_BA8*
Chronic pain	1	4277	46	24	36	R_8C*; R_p9‐46v; R_8Av; R_8Ad; R_i6‐8; R_s6‐8	R_BA8*; R_BA46
	2	1346	−24	18	40	L_8Ad*; L_s6‐8	L_BA8*
	3	837	28	52	18	R_9‐46d*; R_BA46; R_a9‐46v	R_BA9*; R_BA46
	4	1514	−30	48	20	L_9‐46d*; L_BA46	L_BA9*; L_BA46
	5	85	−38	22	50	L_8Av*	L_BA8*
Placebo analgesia	1	1681	44	33	23	R_p9‐46v*; R_IFSp	R_BA46*
	2	941	−34	24	29	L_8C*; L_p9‐46v; L_8Av	L_BA8*; L_BA46
	3	218	30	48	23	R_9‐46d*; R_BA46	R_BA46*
	4	121	37	22	38	R_8C*	R_BA8*
	5	78	−32	30	40	L_8Ad*; L_8Av	L_BA8*
Conditioned pain modulation	1	162	−48	28	24	L_p9‐46v*; L_IFSp	L_BA46*
	2	172	−40	40	12	L_a9‐46v*; L_BA46; L_IFSa	L_BA46*
	3	157	−40	36	40	L_BA46*	L_BA46*
	4	146	40	26	40	R_8C*	R_BA8*
	5	101	56	18	14	R_BA44*	R_BA44*
	6	83	−34	51	28	L_9‐46d*	L_BA9*
Offset analgesia	1	795	−30	10	37	L_8C*; L_IFJa	L_BA8*; L_BA9
	2	685	−52	24	16	L_BA45*; L_IFSp; L_BA44	L_BA45*; L_BA44; L_BA46
Miscellaneous pain modulation	1	1038	−30	42	24	L_9‐46d*; L_BA46; L_9a	L_BA9*; L_BA46
	2	1423	42	10	38	R_8Av*; R_8C	L_BA8*
	3	771	−33	40	44	L_BA46*; L_9‐46d	L_BA46*; L_BA9
	4	466	−40	38	18	L_BA46*; L_p9‐46v	L_BA46*
	5	425	28	30	36	R_8Ad*; R_8Av; R_BA46	R_BA8*; R_BA46
	6	819	48	24	28	R_p9‐46v*; R_BA45; R_IFSp; R_IFJa	R_BA46*; R_BA9; R_BA45
	7	61	46	40	20	R_P9‐46v*	R_BA46*
	8	60	−40	22	32	L_8C*	L_BA8*

*Note:* Occupying dorsolateral prefrontal cortex parcels, as well as grouped Brodmann areas are provided in adjacent cells to cluster volumes. Note that bolded text and “*” are used to delineate within which parcel or Brodmann area the peak of each **ALE** cluster was isolated to, with adjacent parcels and Brodmann areas listed if the significant cluster extended beyond a single subregion.

Abbreviations: BA, Brodmanns Area; L_, Left; R_, Right.

Whilst noxious stimuli in chronic pain patients also evoked signal intensity changes in the left and right dlPFC, unlike changes in pain‐free controls, fewer studies reported dlPFC signal increases (25 of 51: 49%) than reported signal decreases (27 of 51: 53%) (Figure 3). During acute pain stimulation in chronic pain patients, signal changes were bilateral, with signal intensity increases most frequently located in the areas encompassing the left 8ad, 9‐46d, 9p and right 8C, 9‐46d, 8Av and signal intensity decreases in the left and right 8ad, 8C and 8Av. Much like in acute pain, significant ALE clusters in chronic pain studies were found equally represented in both cortical hemispheres, with three in the left and two in the right cortical hemisphere. These ALE clusters occupied BA 8, 9 and 46 (Table 3).

### 3.2 Directionality, Lateralization and Engagement of the dlPFC in Pain Modulatory Phenomenon

**FIGURE 4:**
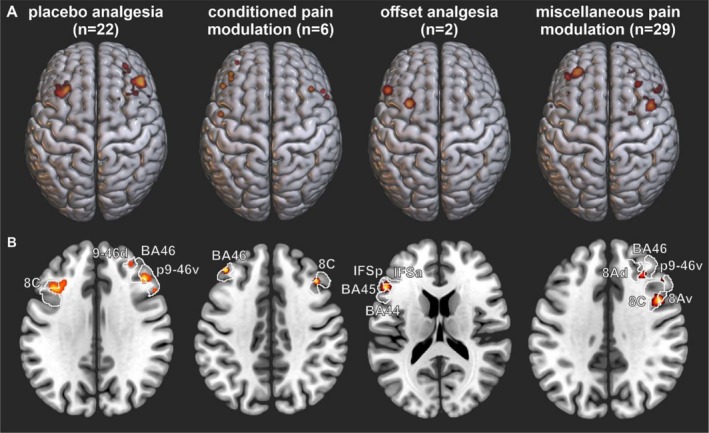
Activation Likelihood Estimate (ALE) pattern masks of dorsolateral prefrontal involvement in endogenous pain modulatory phenomenon. (A) Three main phenomena were chosen for this meta‐analysis: Placebo Analgesia (22 records; 33 foci), Conditioned Pain Modulation (6 records; 8 foci) and Offset Analgesia (2 records; 2 foci). We additionally screened any miscellaneous effects such as in music‐induced or acupuncture analgesia as an additional ALE to provide a more complete dissection of dlPFC engagement across these paradigms (29 records; 43 foci). (B) Of these records, PA appeared to most resemble acute and chronic pain ALE's, with a bilateral involvement despite simulating left side only stimulation, with resulting clusters predominantly occupying Brodmann areas 8, 9 and 46. CPM and OA, whilst limited in foci number for comparison, appeared to predominantly activate the ipsilateral cortical hemisphere with respect to side of stimulation (left). By definition, as we have clustered several different analgesic paradigms under ‘miscellaneous pain modulation’, whilst this ALE produced bilateral results, which is of interest, these clusters were scattered across several dlPFC subregions rather than forming localizations to specific Brodmann areas. Note that these ALE maps were generated at an uncorrected *p*‐value threshold of *p* < 0.001, with a minimum cluster forming volume of 32 mm within Ginger‐ALE software version 3.0.2.

The location of dlPFC regions displaying significant signal intensity changes during pain modulatory phenomenon are shown in Figure 4. For placebo analgesia, the majority of studies (78%) described signal increases in the dlPFC, with signal changes reported on both the left and right sides. Signal increases were most frequently located in the left 9‐46d and 8C and in the right 9‐46d and p9‐46v. In contrast, signal decreases were located most frequently in the left 8C. Significant ALE clusters were also bilateral in nature, with two in the left and three in the right cortical hemisphere, isolated to BA 8 and 46. During CPM paradigms, 3 studies reported signal increases and 3 reported signal decreases, with signal changes most frequently in BA 46. Only 2 studies were identified that assessed offset analgesia, and one performed in individuals with chronic pain reported signal decreases in the left 8C, and the other in pain‐free controls reported signal increases in the right 45. ALE maps generated for both CPM and OA showed hemispheric lateralization, with four and two clusters forming in the left and two and zero clusters forming in the right cortical hemisphere, respectively. Despite this laterality, these findings still demonstrated a greater involvement of BA 8, 9 and 46 in both pain modulatory phenomena (Table 3).

Finally, we assessed dlPFC signal intensity changes during a range of other pain modulatory paradigms including expectancy pain modulation, non‐invasive brain stimulation, attentional analgesia and emotional pain modulation. The majority (72%) reported dlPFC signal intensity increases and these were located most frequently in the left and right 9‐46d and 46, and the right 8Av. Signal decreases were most often reported to occur in the left 8Av and 46. As in acute and chronic pain, no hemispheric lateralization was apparent in the ALE map generated for miscellaneous phenomena, with five and three clusters forming in the left and right hemispheres, respectively (Table 3).

### 3.3 Locations of Signal Change Maxima Across Brodmann's Areas

**FIGURE 5:**
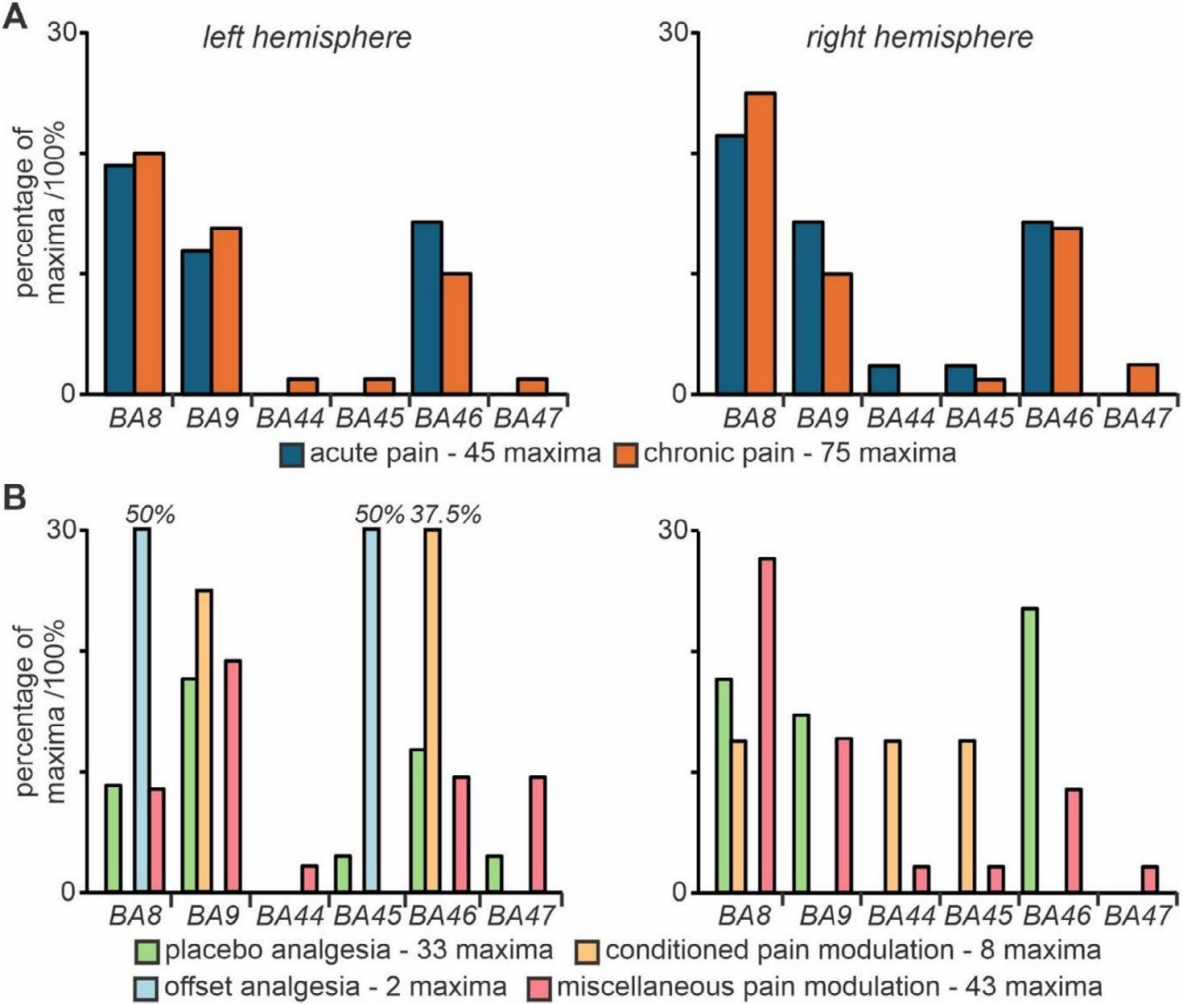
Percentage of local maxima in pain perception and modulation occupying each dorsolateral prefrontal Brodmann area in the left (ipsilateral) and right (contralateral) cortical hemisphere. Through extraction of cluster maximum co‐ordinates in montreal neurological institute space, we conducted a probability analysis designating the percentage of total maxima in both acute (blue bars) and chronic (orange bars) pain (A), and pain modulatory phenomena (B) that occupied each subregion of Brodmann areas which comprise the dorsolateral prefrontal cortex. Percentages are given as a total across the left and right cortical hemispheres to assess laterality in peak activation within each separate subregion of the dlPFC. Notably, very few maxima across acute and chronic pain, and in pain modulatory settings were identified within Brodmann areas 44, 45 and 47. Conversely, predominant activation was observed within the left and right BA8, 9 and 46, associated with both pain perception in an acute and chronic setting, as well as under modulatory effects on pain such as placebo analgesia (green bars) and miscellaneous (pink bars) modulatory phenomena. *BA = Brodmann area*.

We also determined the percentage of maximas in BAs 8–9 and 44–47 (Figure 5). It is clear from the data presented in Figure 5A that acute pain in both pain‐free individuals and in those with chronic pain evoked dlPFC signal intensity changes most frequently in left and right BAs 8, 9 and 46. In contrast, as depicted in Figure 5B, the locations of dlPFC signal intensity changes during various analgesic paradigms were not as consistent. Whilst both placebo analgesia and CPM evoked signal changes primarily in the left and right BAs 8, 9 and 46, offset analgesia evoked changes in exclusively left BAs 8 and 45. Miscellaneous analgesic paradigms were also associated with maximas in BAs 9 and 46, but also in BAs 44 and 45.

## 4 Discussion

### 4.1 dlPFC Involvement in Acute Pain and Pain Modulatory Mechanisms

This study localizes dlPFC activity changes during perceived pain in pain‐free controls and individuals with chronic pain, as well as during various pain modulation paradigms. Our findings extend on previous reviews that describe the dlPFC's role in pain by adding the findings of pain modulatory paradigms and performing activation‐likelihood estimation. Our ALE analysis supports overall spatial consistency in dlPFC activity changes primarily located in BAs 8, 9 and 46, supported by frequency analysis demonstrating localized maxima across relevant studies that are more prevalent within these same dlPFC subregions during both painful stimuli and pain modulatory paradigms.

Brodmann Areas 9 and 46 are dlPFC sub‐regions implicated in a range of functions including emotional regulation (Buhle et al. 2014), driving appropriate behavioural responses (Sallet et al. 2013), attention (Brosnan and Wiegand 2017), value encoding (Sokol‐Hessner et al. 2012) and decision‐making (Rahnev et al. 2016). Indeed, positive visual imagery like viewing a romantic partner can evoke analgesia, alongside altered function within and coupling between the dlPFC and limbic system (Kornelsen et al. 2019; Zhang et al. 2022). Further, neuroimaging studies have consistently demonstrated activation of the dlPFC during tasks involving pain anticipation, distraction and reappraisal, suggesting a role for the dlPFC in both the sensory and affective dimensions of pain (Travassos et al. 2020). Alongside other prefrontal and cingulate areas, the dlPFC forms part of the brain's executive‐attentional network, engaged during the experience of sensory stimuli (Curtis and D’Esposito 2003; Lorenz et al. 2003).

In addition to processing the sensory and emotional aspects of pain, our findings suggest that BAs 8, 9 and 46 are also preferentially involved in pain modulation. Indeed, the dlPFC is considered an interface between cognitive processing and pain (Seminowicz and Davis 2006; Seminowicz and Moayedi 2017). Analgesic paradigms such as placebo and offset analgesia rely heavily on cognitive appraisal, expectation and dynamic evaluation of pain intensity—functions of the dlPFC. Disruption of dlPFC activity can increase tolerance to noxious cold stimuli (Graff‐Guerrero et al. 2005), inhibit placebo analgesia (Krummenacher et al. 2010) and increase pain thresholds in healthy individuals (Borckardt et al. 2007). Additionally, recent studies have also shown that perceived pain variability in healthy controls is associated with dlPFC activity changes (Crawford, Mills, Meylakh, et al. 2023), and that dlPFC activity and connectivity with the brainstem are altered during pain intensity fluctuations in chronic pain (Meylakh et al. 2024). These data highlight that the dlPFC is not only involved in specific analgesic paradigms but also in spontaneous fluctuations in perceived pain. Given its documented role in attention, it is possible that dlPFC‐driven attentional processes contribute to this variability, since directing attention towards or away from a noxious stimulus is a dynamic process that engages cortical regions and can alter pain intensity (Tracey and Mantyh 2007; Villemure and Bushnell 2002).

The results presented here also show the dlPFC plays a central role in the modulation of pain, for example during analgesic paradigms such as placebo and offset analgesia and conditioned pain modulation. Of note is our miscellaneous pain modulation category, which encompassed several emotional and cognitive methodologies to modulate pain; however, in smaller numbers than more typical pain modulatory paradigms. Interestingly, our overall findings indicate that these alternate methods rely on similar dlPFC subregions—BA 8, 9 and 46. However, any specific mechanisms are likely obscured due to our conflation of these records into a single category. The dlPFC is known to play a critical role in maintaining and updating internal representations of goals and expectations (Miller and Cohen 2001). During placebo paradigms when an individual's current experience does not match their expectations, it is thought that the dlPFC modulates regional brain activity in an attempt to match expected‐experienced differentials—that is, drive error‐prediction signals (Alexander and Brown 2018; Pagnini et al. 2023).

### 4.2 Top‐Down Circuits Encoding dlPFC Activity During Pain

But through which pathways and circuits might the dlPFC exert its modulatory influence on pain? Based on recent human functional connectivity and preclinical anatomical studies, it is likely that the dlPFC modulates pain intensity via indirect connections (e.g., via the amygdala and/or hypothalamus) with well‐defined pain modulatory systems such as the midbrain periaqueductal grey (PAG)‐rostral ventromedial medulla (RVM) pathway (De Preter and Heinricher 2024). For instance, recently it was shown that placebo analgesia is associated with altered functional connectivity between the dlPFC, amygdala and lateral PAG (Crawford, Mills, Peek, et al. 2023). Additionally, it is well‐established from preclinical studies that the lateral PAG produces a non‐opioid‐mediated analgesia upon activation (Keay and Bandler 2001). This analgesic response is part of integrated active behavioural responses that aim to remove an individual from danger (Keay and Bandler 2001). Interestingly, a recent human fMRI study reported a somatotopic organization within the lateral PAG during placebo analgesic responses on the body and face (Crawford et al. 2025). This finding suggests that the dlPFC may be capable of influencing the lateral PAG in a somatotopic fashion. Whether analgesic paradigms such as off‐set analgesia and CPM also recruit the PAG‐RVM circuit via projections from the dlPFC remains unclear, though earlier investigations have suggested the PAG‐RVM circuit is not involved in CPM but is involved in mediating off‐set analgesia (Derbyshire and Osborn 2009; Youssef et al. 2016).

### 4.3 Chronic Pain and the dlPFC

Further, there is evidence that reduced analgesic ability is associated with the development of chronic pain. Reduced CPM is found in several chronic pain conditions, including chronic orofacial (King et al. 2009) and back pain (Rabey et al. 2015), is associated with increased postoperative pain (Yarnitsky 2010), and is proposed as a prognostic risk factor for the development of post‐surgical chronic pain (Wilder‐Smith et al. 2010). However, chronic pain patients display placebo effects in clinical trials (Tuttle et al. 2015), and those exploring evoked placebo analgesia in chronic pain sufferers suggest they remain partially in‐tact (Colloca et al. 2020).

Since the dlPFC is fundamentally linked with brainstem pain‐modulatory circuits, it is unsurprising that numerous studies also report structural and functional changes in the dlPFC of chronic pain patients. For example, lower dlPFC grey matter volume has been reported in many chronic pain conditions including irritable bowel syndrome (Seminowicz et al. 2010), complex regional pain syndrome (Erpelding et al. 2016), trigeminal neuropathic pain (Gustin et al. 2011) and chronic low back pain (Seminowicz et al. 2011). Magnetic resonance spectroscopy studies have also reported decreased biochemical markers of neuronal viability in the dlPFC in chronic pain patients (Grachev et al. 2000); while a study using arterial spinal labelling reported increased global dlPFC activity (Youssef et al. 2014). Accompanying these structural changes is altered function within the dlPFC and its cortical connections—showing a mix of increased and decreased functional coupling to other pain processing regions in both neuropathic and musculoskeletal chronic pain states such as chronic low back pain and peripheral neuropathic pain (Jennifer Kornelsen et al. 2013; Verriotis et al. 2022). These changes are likely underpinned by neural damage, although some studies suggest that such losses may be reversible. For example, dlPFC grey matter increases were reported in chronic back pain patients following spinal surgery (Seminowicz et al. 2011), in osteoarthritis pain following joint replacement (Rodriguez‐Raecke et al. 2009), and in complex regional pain syndrome following treatment (Erpelding et al. 2016). Consistent with these dlPFC volumetric reversals and the role of the dlPFC in analgesic system function, it has also been shown that CPM analgesia improves in patients with chronic neck pain following manual therapy that reduced the intensity of on‐going pain (Mata et al. 2024), and following duloxetine treatment in diabetic neuropathic pain (Yarnitsky et al. 2012).

With the dlPFC being critical in pain perception, many studies have begun to non‐invasively target dlPFC activity for pain relief. A recent meta‐analysis of non‐invasive electrical stimulation of the dlPFC in individuals with various forms of chronic pain and in pain‐free controls concluded that whilst dlPFC stimulation did not significantly affect evoked pain in pain‐free individuals, it did show promise in reducing on‐going pain intensity in chronic pain patients (Wu et al. 2024). This result aligns with another recent meta‐analysis that found that dlPFC repetitive transcranial magnetic stimulation was effective overall for chronic pain but not for individuals with chronic neuropathic pain (Zhou et al. 2024). This variability in effect is interesting considering our analysis found that whilst almost all acute pain in pain‐free participant studies reported dlPFC signal increases, we identified chronic pain patients reporting more signal decreases than increases in the dlPFC. Whether similar effects also occur during analgesic paradigms is unknown; however, it does appear that although the dlPFC shows signal change in both pain‐free and chronic pain patients, it is likely that the underlying neural changes are different.

### 4.4 Limitations

Some limitations must be considered in the findings generated by this review. Primarily, we did not conduct a typical risk of bias assessment of included studies, as the focus of the present investigation was not to assess literature quality but determine overlapping and disparate dlPFC activation maxima within and between imaging studies assessing pain perception and modulation. We did however conduct a modified ROBINS‐I risk of bias summary, which indicated only two notable potential high risks of bias largely confined to records assessing chronic pain states. Given that in experimental human brain imaging studies of chronic pain, it is field standard to compare against a pain‐free control cohort, the lack of randomization procedures in these domains may conflate pain‐specific findings with aetiological changes in the patient group inherently absent from the control comparator, raising potential risks of bias. Moreover, reporting on actual or potential adverse events was largely absent from these records, where in conditions such as chronic pain one would expect the likelihood of adverse events related to being confined within an MRI scanner to be generally greater. We elected not to remove any records from our analysis based on these risk of bias findings, since all included records returned low to moderate risk in terms of experimental design, and all performed appropriate statistical thresholds for functional imaging assessments (i.e., never above *p* < 0.005 uncorrected), and had reasonable sample sizes for making these assessments (mean *n* = 36), with GingerALE adjusting grouped cluster sizes based on sample size heterogeneity between studies. Additionally, a limitation of the current approach is that our search string captured both task‐activated and resting connectivity changes involving the dlPFC in both pain and pain modulatory settings. This approach enabled us to provide a general summary of which regions of the dlPFC are consistently associated with these settings across functional imaging modalities; however, our findings should be acknowledged in this light since neural activity profiles assessed through adherence to a general linear model versus a seed timeseries conceptually differ. Despite this conflation, our findings indicate a consistent overlap between our six settings of Brodmann areas 8, 9 and 46 of the dlPFC as being most commonly involved, suggesting that this conceptual merging does not detract from any between‐study effects. Moreover, our search strings were also limited such that articles were only included with ‘dorsolateral prefrontal cortex’ as a core concept. It should be considered that often articles do not include specific brain regions within the title, abstract, or keywords and rather brain networks or systems are included instead. Here, emphasis was placed on capturing the largest number of articles that focused on pain perception or modulation, as well as dlPFC functional changes. With that in mind, the search we conducted and findings we present may underestimate the total literature tying cortical activation within the dlPFC to either pain perception or modulation. By taking a more stringent approach with our elected search terms, we ensured that we solely focus this review on experimental findings which highlight the role of the dlPFC in pain states or associated with endogenous analgesic phenomena.

## 5 Conclusions

As expected, we found altered dlPFC signal intensity and connectivity during acute pain in pain‐free controls and individuals with chronic pain. Furthermore, we found dlPFC signal intensity and connectivity changes during various analgesic paradigms. Importantly, these changes were largely restricted to BAs 8, 9 and 46, brain regions with well‐established roles in emotional regulation, driving appropriate behavioural responses, attention, value encoding and decision‐making and have been shown in previous studies to be altered in individuals with chronic pain. These findings underpin an important role for the dlPFC in both acute pain perception and modulation and in the pathophysiology of chronic pain.

## Author Contributions

This study was designed by L.S.C.; K.A.K.; and L.A.H. Data were collected by L.S.C.; D.C.B.; and J.W.M.K. Data analyses were performed by L.S.C. and A.P. All authors equally provided critical analysis of the primary findings; and initial manuscript construction was performed by L.S.C. D.C.B.; J.W.M.K.; and A.P. conducted manuscript editing. All authors approved the final manuscript, and provided agreement of accountability for all aspects of the work.

## Funding

This work was supported by the National Health and Medical Research Council of Australia (Grant 1130280).

## Disclosure

No generative artificial intelligence (AI) or AI‐assisted technologies were used in the preparation of this manuscript.

## Conflicts of Interest

The authors declare no conflicts of interest.

## Supporting information


**Data S1:** ejp70318‐sup‐0001‐Tables.docx.
**Table S1:** List of studies assessed to meet the criteria for inclusion in this review. Each included study is listed by concept. Extracted data included: DOI; author and date of publication; pain category; the side of stimulation applied (if applicable); the primary dorsolateral prefrontal cortex (dlPFC) parcel within the left or right cortical hemisphere activated; and the specific pain condition or modulatory phenomenon of interest. Sample size is included with brackets denoting healthy controls if two participant groups were compared to produce the dlPFC cluster maxima. (a): activity changes; (b): functional connectivity changes.
**Table S2:** Structured risk of bias summary table for each included study following a modified version of the Risk of Bias in Non‐randomized Studies—of Interventions (ROBINS‐I) framework. Six aspects were assessed, specifically, risk of bias arising from: study design; randomization procedures; blinding of outcome assessment; withdrawal and drop out reporting; listed inclusion/exclusion criteria; and reporting on adverse events. Green shading/‘LOW’ = low risk of bias, Yellow shading/‘MEDIUM’ = medium risk of bias, Red shading/‘HIGH’ = high risk of bias.

## Data Availability

All software used to generate results presented in this report is publicly available, and can be downloaded for free from the following sources: GingerALE—https://www.brainmap.org/ale/; MRICROGL—https://www.nitrc.org/projects/mricrogl/.
