# Supplementary material for: The Human Dorsolateral Prefrontal Cortex in Pain and Pain Modulation: A Review and Activation‐Likelihood Estimation Approach
Source: Eur J Pain. 2026 Jun 24;30(6):e70318. doi: 10.1002/ejp.70318 (PMC13292397; doi:10.1002/ejp.70318)
Supplement: Supplementary file 1 — Data S1: ejp70318‐sup‐0001‐Tables.docx. Table S1: List of studies assessed to meet the criteria for inclusion in this review. Each included study is listed by concept. Extracted data included: DOI; author and date of publication; pain category; the side of stimulation applied (if applicable); the primary dorsolateral prefrontal cortex (dlPFC) parcel within the left or right cortical hemisphere activated; and the specific pain condition or modulatory phenomenon of interest. Sample size is included with brackets denoting healthy controls if two participant groups were compared to produce the dlPFC cluster maxima. (a): activity changes; (b): functional connectivity changes. Table S2: Structured risk of bias summary table for each included study following a modified version of the Risk of Bias in Non‐randomized Studies—of Interventions (ROBINS‐I) framework. Six aspects were assessed, specifically, risk of bias arising from: study design; randomization procedures; blinding of outcome assessment; withdrawal and drop out reporting; listed inclusion/exclusion criteria; and reporting on adverse events. Green shading/‘LOW’ = low risk of bias, Yellow shading/‘MEDIUM’ = medium risk of bias, Red shading/‘HIGH’ = high risk of bias. [file EJP-30-0-s001.docx]

**Table 1:** List of studies assessed to meet the criteria for inclusion in this review. Each included study is listed by concept. Extracted data included: DOI; author and date of publication; pain category; the side of stimulation applied (if applicable); the primary dorsolateral prefrontal cortex (dlPFC) parcel within the left or right cortical hemisphere activated; and the specific pain condition or modulatory phenomenon of interest. Sample size is included with brackets denoting healthy controls if two participant groups were compared to produce the dlPFC cluster maxima. *(a): activity changes; (b): functional connectivity changes.*

| **DOI** | | | | **authors (y)** | | | | **side of stimulation** | | **primary left dlPFC region locality** | **primary right dlPFC region locality** | **dlPFC direction of change** | **condition of interest** | **sample size (+HC)** |
| --- | --- | --- | --- | --- | --- | --- | --- | --- | --- | --- | --- | --- | --- | --- |
| **acute pain** |  | | | | | | |  | |  |  |  |  |  |
| 10.1016/j.neuroimage.2010.11.061 | | | | Maihofner et al., 2011 | | | | left | | 8C | 8C | increase (a) | experimental \| healthy control only | (12) |
| 10.3389/fnagi.2020.00116 | | | | Gonzalez-Roldan et al., 2020 | | | | left | |  | 46 | increase (b) | experimental \| healthy control only | (75) |
| 10.1097/j.pain.0000000000000923 | | | | Forkmann et al., 2017 | | | | left | | 9-46d | 8Av | decrease (a) | experimental \| parkinsons disease | 21 (23) |
| 10.1001/archpsyc.65.11.1275 | | | | Strigo et al., 2008 | | | | left | | 8C | I6-8 | increase (a) | experimental \| major depressive disorder | 15 (15) |
| 10.1093/cercor/bhac332 | | | | Crawford et al., 2023 | | | | right | | P9-46v | P47r | increase (a) | experimental \| healthy control only | (63) |
| 10.1523/jneurosci.5538-08.2009 | | | | Oshiro et al., 2009 | | | | left | | 8C | 46 | increase (a) | experimental \| healthy control only | (12) |
| 10.1523/jneurosci.5128-06.2007 | | | | Oshiro et al., 2007 | | | | left | | A47r | 46 | increase (a) | experimental \| healthy control only | (12) |
| 10.1016/j.pain.2014.05.015 | | | | Atlas et al., 2014 | | | | left | | 46 | 46 | increase (a) | experimental \| healthy control only | (30) |
| 10.1016/j.pain.2005.11.008 | | | | Seminowicz et al., 2006 | | | | left | | 8Ad | 8Ad | increase (a) | experimental \| healthy control only | (22) |
| 10.1093/brain/awf137 | | | | Bornhovd et al., 2002 | | | | left | | IFJa | IFJp | increase (a) | experimental \| healthy control only | (9) |
| 10.1080/08990220902738243 | | | | Freund et al., 2009 | | | | bilateral | | 9-46d | IFJa | increase (a) | experimental \| healthy control only | (15) |
| 10.3389/fnins.2023.1234286 | | | | Borelli et al., 2023 | | | | left | |  | 45 | increase (a) | experimental \| healthy control only | (34) |
| 10.1016/j.neuroimage.2007.02.001 | | | | Moulton et al., 2007 | | | | left | | 8Av | P9-46v | increase (a) | experimental \| healthy control only | (12) |
| 10.7554/eLife.97793 | | | | Wittkamp et al., 2024 | | | | left | | 8Av |  | decrease (a) | experimental \| healthy control only | (50) |
| 10.1016/j.pain.2005.01.005 | | | | Zambreanu et al., 2005 | | | | not reported | |  | 9a | increase (a) | experimental \| healthy control only | (12) |
| 10.1097/j.pain.0000000000002814 | | | | Peng et al., 2023 | | | | left | | 46 |  | increase (b) | experimental \| healthy control only | (60) |
| 10.1002/j.1532-2149.2011.00027.x | | | | Benson et al., 2012 | | | | bilateral | | IFJp |  | increase (a) | experimental \| healthy control only | (30) |
| 10.1523/jneurosci.0006-12.2012 | | | | Moulton et al., 2012 | | | | right | | IFJa |  | increase (a) | experimental \| healthy control only | (16) |
| 10.1002/hbm.22035 | | | | Seifert et al., 2013 | | | | left | | 9p | 8BL | increase (a) | experimental \| healthy control only | (9) |
| 10.1016/j.neures.2011.01.015 | | | | Uematsu et al., 2011 | | | | right | | p9-46v | p9-46v | increase (a) | experimental \| healthy control only | (17) |
| 10.1016/j.neuroimage.2019.01.039 | | | | Su et al., 2019 | | | | right | |  | 46 | increase (a) | experimental \| healthy control only | (51) |
| 10.1523/jneurosci.1954-15.2016 | | | | Bräscher et al., 2016 | | | | not reported | |  | 9-46d \| 8C | decrease (a) | experimental \| healthy control only | (23) |
| 10.1016/j.pain.2008.10.027 | | | | Veldhuijzen et al., 2009 | | | | left | | 46 |  | increase (a) | experimental \| healthy control only | (10) |
| 10.1016/j.pain.2004.02.033 | | | | Valet et al., 2004 | | | | right | | 9-46d | 9-46d | Increase (b) | experimental \| healthy control only | (7) |
| 10.1016/j.pain.2013.06.041 | | | | Tseng et al., 2013 | | | | right | | i6-8 | a9-46v | Increase (a) | experimental \| healthy control only | (23) |
| 10.1002/hbm.24824 | | | | Letzen et al., 2020 | | | | left | |  | 8Av | Increase (b) | experimental \| healthy control only | (22) |
| 10.1073/pnas.1511269112 | | | | Rütgen et al., 2015 | | | | left | |  | 9-46d | Increase (a) | experimental \| healthy control only | (102) |
| ***chronic pain*** | | |  | | | | |  | |  |  |  |  |  |
| 10.1371/journal.pone.0156545 | | | | Hubbard et al., 2016 | | | | bilateral | | 8C | 8C | decrease (b) | irritable bowel syndrome | 19 (19) |
| 10.1177/0022034521994089 | | | | Lim et al., 2021 | | | | right | |  | p9-46v | decrease (b) | temporomandibular disorder | 12 (24) |
| 10.1371/journal.pone.0221023 | | | | Ihara et al., 2019 | | | | bilateral | | 9a | 46 | increase (b) | chronic neck pain | 20 (20) |
| 10.1002/art.41220 | | | | Hubbard et al., 2020 | | | | left | |  | 8C | increase (a) | fibromyalgia | 38 (15) |
| 10.2147/jpr.S327945 | | | | Zhu et al., 2021 | | | | bilateral | |  | 9-46d | increase (b) | episodic and chronic migraine | 56 (35) |
| 10.1186/s10194-021-01381-w | | | | Mungoven et al., 2022 | | | | right | | p9-46v |  | increase (a) | episodic migraine | 25 (29) |
| 10.1111/head.14360 | | | | Szabo et al., 2022 | | | | bilateral | | 9-46d |  | decrease (b) | chronic headache | 13 (13) |
| 10.1016/j.ejpn.2022.10.004 | | | | Szabo et al., 2022 | | | | bilateral | | 8Ad |  | decrease (b) | endometriosis | 11 (14) |
| 10.1097/j.pain.0000000000000951 | | | | Wang et al., 2017 | | | | bilateral | | 8Ad | S6-8 | increase (b) | trigeminal neuralgia | 38 (38) |
| 10.1097/psy.0000000000000655 | | | | Bhatt et al., 2019 | | | | bilateral | | 8C |  | decrease (b) | irritable bowel syndrome | 32 (26) |
| 10.1016/j.bbi.2019.06.024 | | | | Sandstrom et al., 2019 | | | | left | | 8Ad | 8Av | decrease (a) | rheumatoid arthritis | 31 (23) |
| 10.1097/wnr.0000000000000529 | | | | Wang et al., 2016 | | | | bilateral | |  | p9-46v | increase (b) | migraine | 34 (25) |
| 10.1007/s00234-020-02582-x | | | | Dai et al., 2021 | | | | bilateral | | a9-46v | p9-46v | decrease (b) | chronic migraine | 17 (35) |
| 10.1186/s10194-016-0610-4 | | | | Wang et al., 2016 | | | | bilateral | |  | 8C | increase (b) | migraine | 20 (25) |
| 10.3389/fpain.2022.918766 | | | | Verriotis et al., 2022 | | | | bilateral | |  | a9-46v | decrease (b) | chronic peripheral neuropathic pain | 17 (17) |
| 10.1007/s00540-017-2343-1 | | | | Matsuo et al., 2017 | | | | left | | 8Ad | 8C | decrease (a) | chronic low back pain | 11 (13) |
| 10.1093/cercor/bhp205 | | | | Gustin et al., 2010 | | | | bilateral | |  | 9-46d | increase (a) | spinal cord injury | 23 (45) |
| 10.1002/j.1532-2149.2011.00058.x | | | | Burgmer et al., 2012 | | | | right | | p47r |  | decrease (a) | fibromyalgia | 17 (17) |
| 10.1016/j.pain.2013.11.008 | | | | Youssef et al., 2014 | | | | bilateral | | 9-46d |  | increase (a) | painful trigeminal neuralgia + temporomandibular disorder | 33 (54) |
| 10.1186/s10194-017-0747-9 | | | | Chen et al., 2017 | | | | bilateral | | 9-46d | 45 | decrease (b) | episodic migraine | 18 (18) |
| 10.1016/j.jpain.2023.05.006 | | | | Sandstrom et al., 2023 | | | | bilateral | |  | 8Ad | decrease (a) | fibromyalgia | 65 (33) |
| 10.3389/fnhum.2016.00489 | | | | Mathur et al., 2016 | | | | Left | | IFSp |  | decrease (a) | migraine | 14 (14) |
| 10.1097/j.pain.0000000000001907 | | | | Sandstrom et al., 2020 | | | | Left | | IFJa |  | increase (a) | fibromyalgia | 67 (34) |
| 10.1016/j.nicl.2023.103355 | | | | Mosch et al., 2023 | | | | Left | | 8Av |  | decrease (a) | fibromyalgia | 23 (21) |
| 10.1093/brain/awh098 | | | | Gracely et al., 2004 | | | | Left | | 8Av |  | increase (a) | fibromyalgia | 29 (0) |
| 10.1002/hbm.22757 | | | | Ceko et al., 2015 | | | | Bilateral | | p9-46v | p9-46v | decrease (b) | chronic low back pain | 14 (16) |
| 10.1002/hbm.25401 | | | | Huynh et al., 2021 | | | | Bilateral | |  | i6-8 | decrease (b) | paraplegic chronic pain | 29 (25) |
| 10.1016/j.neulet.2014.08.027 | | | | Hiramatsu et al., 2014 | | | | right | | 9p | 8Av | increase (b) | osteoarthritis | 12 (11) |
| https://doi.org/10.1111/head.14900 | | | | Zhao et al., 2025 | | | | bilateral | |  | 9p | increase (b) | chronic headache | 33 (30) |
| 10.1523/eneuro.0522-23.2024 | | | | Meylakh et al., 2024 | | | | left | | 9-46d |  | increase (b) | painful trigeminal neuralgia | 24 |
| 10.2147/jpr.S500924 | | | | Gao et al., 2025 | | | | bilateral | | 8Ad | 8Ad | increase/ decrease (b) | chronic neck pain | 89 (86) |
| 10.21037/qims-23-1687 | | | | Jin et al., 2024 | | | | bilateral | | 9p |  | increase (b) | primary dysmenorrhea | 25 (20) |
| 10.1007/s40122-022-00365-1 | | | | Cao et al., 2022 | | | | bilateral | | 46 | 8Ad | decrease (b) | migraine | 30 (40) |
| 10.1111/pme.12267 | | | | Schwedt et al., 2014 | | | | bilateral | | 46 | 9p | decrease (a) | migraine | 38 (28) |
| 10.23736/s1973-9087.24.08087-0 | | | | Zhang et al., 2024 | | | | bilateral | | 46 | a9-46v | increase (b) | chronic low back pain | 16 |
| 10.1016/j.neuroimage.2008.09.008 | | | | Burgmer et al., 2009 | | | | right | |  | 8Av | increase (a) | fibromyalgia | 18 (19) |
| 10.1016/j.clinph.2014.10.004 | | | | Ma et al., 2015 | | | | bilateral | |  | 8Av | decrease (b) | irritable bowel syndrome | 21 (21) |
| 10.1002/ana.22537 | | | | Mainero et al., 2011 | | | | bilateral | |  | a9-46v | decrease (b) | migraine | 17 (17) |
| 10.1016/j.brainres.2021.147608 | | | | Tang et al., 2021 | | | | bilateral | |  | 9-46d | decrease (b) | viral neuralgia | 25 (21) |
| 10.1371/journal.pone.0004542 | | | | Cauda et al., 2009 | | | | bilateral | | 9-46d | 8C | decrease (b) | diabetic neuropathy | 8 (8) |
| 10.1002/hbm.25038 | | | | Mao et al., 2020 | | | | bilateral | | IFJp |  | increase (b) | irritable bowel syndrome | 34 (34) |
| 10.1002/hbm.23696 | | | | Tsai et al., 2018 | | | | bilateral | | s6-8 | 8Av | decrease (b) | painful trigeminal neuralgia | 64 (19) |
| 10.1097/j.pain.0000000000002933 | | | | Tong et al., 2023 | | | | left | |  | 9-46d | increase (a) | fibromyalgia | 32 |
| 10.1111/j.1365-2982.2005.00720.x | | | | Andresen et al., 2005 | | | | bilateral | |  | IFSp | increase (a) | irritable bowel syndrome | 8 (8) |
| 10.1002/acr.23601 | | | | Lee et al., 2019 | | | | left | | 8Ad | s6-8 | increase (a) | rheumatoid arthritis | 14 (16) |
| 10.1515/sjpain-2018-0341 | | | | Kornelsen et al., 2013 | | | | bilateral | |  | IFJp \| 9-46d | increase (a) | failed back surgery syndrome | 14 (14) |
| 10.1186/s10194-020-01210-6 | | | | Lim et al., 2021 | | | | bilateral | |  | 8C | decrease (b) | migraine | 20 (26) |
| 10.3389/fnhum.2018.00107 | | | | Zhang et al., 2018 | | | | right | | 8Av |  | decrease (b) | painful trigeminal neuralgia | 29 (34) |
| 10.1097/j.pain.0000000000000633 | | | | Schrepf et al., 2016 | | | | left | | 8C |  | decrease (b) | fibromyalgia | 18 |
| 10.1177/0333102414526069 | | | | Schwedt et al., 2014 | | | | left | |  | IFJa | increase (a) | migraine | 24 (27) |
| 10.1097/j.pain.0000000000001366 | | | | Roy et al., 2018 | | | | right | |  | 46 | increase (a) | chronic jaw pain | 16 (15) |
| ***placebo analgesia*** | | | | |  | | |  | |  |  |  |  |  |
| 10.3389/fnbeh.2021.696577 | | | | Shi et al., 2021 | | | | right | | p47r |  | decrease (a) | experimental \| healthy control only | (20) |
| 10.1016/j.pain.2012.08.008 | | | | Hashmi et al., 2012 | | | | bilateral | | 8Av |  | increase (b) | experimental \| chronic low back pain | 30 |
| 10.1016/j.jpain.2015.05.001 | | | | Sevel et al., 2015 | | | | bilateral | | 9-46d | 9-46d | increase (b) | experimental \| healthy control only | (24) |
| 10.1093/cercor/bhad247 | | | | Crawford et al., 2023 | | | | right | |  | 46 | increase (a) | experimental \| healthy control only | (38) |
| 10.1016/j.pain.2013.03.031 | | | | Huber et al., 2013 | | | | bilateral | |  | IFSp | increase (a) | experimental \| healthy control only | (36) |
| 10.1073/pnas.2101273118 | | | | Tu et al., 2021 | | | | right | |  | 45 | increase (b) | experimental \| healthy control only | (81) |
| 10.1016/j.pain.2011.10.036 | | | | Elsenbruch et al., 2012 | | | | bilateral | | 8C |  | decrease (a) | experimental \| healthy control only | (36) |
| 10.1155/2020/8820443 | | | | Shi et al., 2020 | | | | right | | 9p | 46 | decrease (b) | experimental \| healthy control only | (30) |
| 10.1016/j.neuroimage.2015.01.056 | | | | Sevel et al., 2015 | | | | bilateral | | p9-46v | p9-46v | increase (b) | experimental \| healthy control only | (52) |
| 10.1016/j.pain.2009.04.003 | | | | Watson et al., 2009 | | | | right | | 9-46d |  | increase (a) | experimental \| healthy control only | (11) |
| 10.1016/j.nicl.2014.09.007 | | | | Schmidt-Wilcke et al., 2014 | | | | left | | 8C |  | decrease (b) | experimental \| fibromyalgia | 15 |
| 10.1016/j.neuroimage.2019.116510 | | | | Schenk & Colloca., 2020 | | | | not reported | |  | 8C | increase (b) | experimental \| healthy control only | (38) |
| 10.1093/scan/nsx033 | | | | Van Der Meulen et al., 2017 | | | | left | | 8C | 8Av | increase (a) | experimental \| healthy control only | (30) |
| 10.1111/nmo.12454 | | | | Theysohn et al., 2014 | | | | bilateral | |  | 9-46d | increase (a) | experimental \| healthy control only | (30) |
| 10.1038/s41598-024-77693-z | | | | Wolf et al., 2024 | | | | left | |  | 8Av | increase (a) | experimental \| healthy control only | (41) |
| 10.1016/j.neuron.2009.07.014 | | | | Eippert et al., 2009 | | | | left | | 9-46d | 8Ad | increase (a) | experimental \| healthy control only | (48) |
| 10.1523/jneurosci.3556-05.2006 | | | | Kong et al., 2006 | | | | right | | IFJa | p9-46v | increase (a) | experimental \| healthy control only | (24) |
| 10.1016/j.neuroimage.2012.11.029 | | | | Geuter et al., 2013 | | | | left | |  | p9-46v | increase (a) | experimental \| healthy control only | (52) |
| 10.1523/jneurosci.2542-15.2015 | | | | Zeidan et al., 2015 | | | | right | | 8C | 8Ad | increase (a) | experimental \| healthy control only | (75) |
| 10.1016/j.pain.2010.09.021 | | | | Lui et al., 2010 | | | | bilateral | |  | p9-46v | increase (a) | experimental \| healthy control only | (31) |
| 10.1038/s41598-017-18870-1 | | | | Makary et al., 2018 | | | | bilateral | | p9-46v | IFSp | decrease (a) | experimental \| healthy control only | (19) |
| 10.1111/pme.12783 | | | | Fehse et al., 2015 | | | | left | |  | p9-46v | increase (a) | experimental \| healthy control only | (30) |
| ***conditioned pain modulation*** | | | | | |  | |  | |  |  |  |  |  |
| 10.1097/pr9.0000000000000999 | | | | Harrison et al., 2022 | | | | right | |  | IFJp | increase (b) | experimental \| healthy control only | (35) |
| 10.1002/hbm.23199 | | | | Youssef et al., 2016 | | | | right | |  | p9-46v | decrease (a) | experimental \| healthy control only | (54) |
| 10.1016/j.brainres.2011.11.056 | | | | Moont et al., 2012 | | | | left | |  | 8C | decrease (a) | experimental \| healthy control only | (30) |
| 10.1016/j.pain.2006.06.017 | | | | Song et al., 2006 | | | | left | | 46 | 44 | increase (a) | experimental \| healthy control only | (12) |
| 10.1016/j.neuroimage.2021.118742 | | | | Huynh et al., 2022 | | | | bilateral | | 46 |  | decrease (b) | experimental \| healthy control only | (40) |
| 10.1016/j.pain.2014.07.008 | | | | Nahman-Averbuch et al., 2014 | | | | right | |  | 45 | increase (a) | experimental \| healthy control only | (13) |
| ***Offset analgesia*** | |  | | | | | |  | |  |  |  |  |  |
| 10.1177/1744806918767512 | | | | Zhang et al., 2018 | | | | left | | 8C |  | decrease (a) | experimental \| chronic pain | 17 (17) |
| 10.1016/j.pain.2014.07.008 | | | | Nahman-Averbuch et al., 2014 | | | | right | |  | 45 | increase (a) | experimental \| healthy control only | (13) |
| ***miscellaneous pain modulation*** | | | | | | |  | |  |  |  |  |  |  |
| 10.1097/j.pain.0000000000002814 | | | | Peng et al., 2023  (non-invasive brain stimulation) | | | | left | | 46 |  | decrease (b) | experimental \| healthy control only | (60) |
| 10.1523/jneurosci.2568-06.2006 | | | | Weich et al., 2006  (expectancy pain modulation) | | | | left | | 9-46d | 46 | increase (a) | experimental \| healthy control only | (12) |
| 10.1002/ejp.696 | | | | Choi et al., 2016  (TENS analgesia) | | | | left | |  | i6-8 | increase (a) | experimental \| healthy control only | (24) |
| 10.1097/j.pain.0000000000001599 | | | | Lopez-Sola et al., 2019 (emotional pain modulation) | | | | left | |  | p9-46v | decrease (a) | experimental \| healthy control only | (30) |
| 10.1371/journal.pone.0110654 | | | | Wiech et al., 2014 (emotional pain modulation) | | | | left | | 9a | 9-46d | increase (a) | experimental \| healthy control only | (12) |
| 10.1093/scan/nsad018 | | | | Silvestrini et al., 2023 (attentional analgesia) | | | | right | | 8Av | 8C | decrease (b) | experimental \| healthy control only | (29) |
| 10.3389/fnins.2020.00006 | | | | Henderson et al., 2020 (expectancy pain modulation) | | | | right | | a47r | 9-46d | increase (a) | experimental \| healthy control only | (24) |
| 10.1038/s41598-019-51990-4 | | | | Pando-Naude et al., 2019 (music analgesia) | | | | bilateral | |  | p9-46v \| 8Ad | increase (b) | experimental \| fibromyalgia | 20 (20) |
| 10.1002/j.1532-2149.2011.00039.x | | | | Godinho et al., 2012 (emotional pain modulation) | | | | bilateral | | 8BL | 8BL | increase (a) | experimental \| healthy control only | (18) |
| 10.1016/j.jpain.2014.07.006 | | | | Dobek et al., 2014 (music analgesia) | | | | right | | 9-46d | 46 | increase (a) | experimental \| healthy control only | (12) |
| 10.1016/j.neuroimage.2009.05.031 | | | | Vanhaudenhuyse et al., 2009 (hypnosis analgesia) | | | | left | |  | 9-46d | decrease (a) | experimental \| healthy control only | (13) |
| 10.1002/hbm.20716 | | | | Raij et al., 2009 (expectancy pain modulation) | | | | left | |  | 9-46d | increase (a) | experimental \| healthy control only | (14) |
| 10.1186/1744-8069-7-45 | | | | Shukla et al., 2011 (acupuncture analgesia) | | | | left | | 9-46d | IFJp | decrease (a) | experimental \| healthy control only | (10) |
| 10.1089/brain.2016.0440 | | | | Sankarasubramanian et al., 2017 (non-invasive brain stimulation) | | | | left | |  | i6-8 | increase (b) | experimental \| healthy control only | (10) |
| 10.1007/s11682-018-9875-3 | | | | Kong et al., 2019 (exercise analgesia) | | | | bilateral | | 46 | 8Av | increase (b) | fibromyalgia | 21 (20) |
| 10.1111/j.1365-2982.2007.00908.x | | | | Dunckley et al., 2007 (attentional analgesia) | | | | bilateral | |  | 44 | decrease (a) | experimental \| healthy control only | (12) |
| 10.1155/2015/210120 | | | | Shi et al., 2015 (acupuncture analgesia) | | | | bilateral | | 46 |  | increase (b) | experimental \| healthy control only | (28) |
| 10.1093/pm/pnaa178 | | | | Berry et al., 2020 (emotional pain modulation) | | | | left | |  | 8Av | increase (a) | chronic low back pain | 20 |
| 10.1016/j.ejpain.2011.07.010 | | | | Mohr et al., 2012 (expectancy pain modulation) | | | | left | | IFJp | 8C | increase (a) | experimental \| healthy control only | (15) |
| 10.1016/j.nicl.2018.01.021 | | | | Kong et al., 2018 (expectancy pain modulation) | | | | bilateral | | IFJa |  | increase (b) | knee osteoarthritis | 19 |
| 10.3390/brainsci6010008 | | | | Ellingson et al., 2016 (exercise analgesia) | | | | left | |  | 9-46d | increase (a) | fibromyalgia | 12 (12) |
| 10.1523/jneurosci.2542-15.2015 | | | | Zeidan et al., 2015 (emotional pain modulation) | | | | right | | 8Av |  | decrease (a) | experimental \| healthy control only | (17) |
| 10.1038/npp.2013.13 | | | | Taylor et al., 2013 (non-invasive brain stimulation) | | | | right | |  | 8Ad | decrease (a) | experimental \| healthy control only | (14) |
| 10.1016/j.neuroimage.2020.117548 | | | | Oliva et al., 2021 (attentional analgesia) | | | | left | |  | IFSp \| p9-46v | increase (b) | experimental \| healthy control only | (20) |
| 10.1186/s10194-018-0924-5 | | | | Naegel et al., 2018 (non-invasive brain stimulation) | | | | right | |  | i6-8 \| IFSa | increase (a) | experimental \| healthy control only | (13) |
| 10.1038/s41598-022-21557-x | | | | Argaman et al., 2022 (non-invasive brain stimulation) | | | | bilateral | |  | 8Av | increase (b) | fibromyalgia | 27 |
| 10.1523/jneurosci.2123-21.2022 | | | | Zhang et al., 2022 (emotional pain modulation) | | | | right | |  | 8Ad | increase (b) | experimental \| healthy control only | (34) |
| 10.1515/sjpain-2018-0341 | | | | Kornelsen et al., 2019 (emotional pain modulation) | | | | right | | 9a | 44 | increase (b) | experimental \| healthy control only | (32) |
| 10.1371/journal.pone.0013309 | | | | Younger et al., 2010 (emotional pain modulation) | | | | left | |  | 45 | increase (a) | experimental \| healthy control only | (15) |

**Table 2:** Structured risk of bias summary table for each included study following a modified version of the Risk of Bias in Non-randomized Studies – of Interventions (ROBINS-I) framework. Six aspects were assessed, specifically, risk of bias arising from: study design; randomization procedures; blinding of outcome assessment; withdrawal and drop out reporting; listed inclusion/exclusion criteria; and reporting on adverse events. *Green shading / “LOW” = low risk of bias, Yellow shading / “MEDIUM” = medium risk of bias, Red shading / “HIGH” = high risk of bias.*

| **DOI** | | | | **authors (y)** | | | | **Study design** | | **Randomization procedure** | | **Blinding of outcome assessment** | | **Withdrawal and drop out reporting** | | **Inclusion / exclusion criteria** | | **Reporting of adverse events** | |
| --- | --- | --- | --- | --- | --- | --- | --- | --- | --- | --- | --- | --- | --- | --- | --- | --- | --- | --- | --- |
| **acute pain** |  | | | | | | |  | |  | |  | |  | |  | |  | |
| 10.1016/j.neuroimage.2010.11.061 | | | | Maihofner et al., 2011 | | | | LOW | | LOW | | MEDIUM | | LOW | | MEDIUM | | MEDIUM | |
| 10.3389/fnagi.2020.00116 | | | | Gonzalez-Roldan et al., 2020 | | | | LOW | | HIGH | | MEDIUM | | LOW | | LOW | | MEDIUM | |
| 10.1097/j.pain.0000000000000923 | | | | Forkmann et al., 2017 | | | | LOW | | LOW | | MEDIUM | | LOW | | LOW | | MEDIUM | |
| 10.1001/archpsyc.65.11.1275 | | | | Strigo et al., 2008 | | | | LOW | | MEDIUM | | MEDIUM | | LOW | | LOW | | MEDIUM | |
| 10.1093/cercor/bhac332 | | | | Crawford et al., 2023 | | | | LOW | | MEDIUM | | MEDIUM | | LOW | | MEDIUM | | MEDIUM | |
| 10.1523/jneurosci.5538-08.2009 | | | | Oshiro et al., 2009 | | | | LOW | | LOW | | MEDIUM | | LOW | | MEDIUM | | MEDIUM | |
| 10.1523/jneurosci.5128-06.2007 | | | | Oshiro et al., 2007 | | | | LOW | | LOW | | MEDIUM | | MEDIUM | | MEDIUM | | MEDIUM | |
| 10.1016/j.pain.2014.05.015 | | | | Atlas et al., 2014 | | | | LOW | | MEDIUM | | MEDIUM | | LOW | | LOW | | MEDIUM | |
| 10.1016/j.pain.2005.11.008 | | | | Seminowicz et al., 2006 | | | | LOW | | MEDIUM | | MEDIUM | | LOW | | LOW | | MEDIUM | |
| 10.1093/brain/awf137 | | | | Bornhovd et al., 2002 | | | | LOW | | LOW | | MEDIUM | | LOW | | MEDIUM | | MEDIUM | |
| 10.1080/08990220902738243 | | | | Freund et al., 2009 | | | | LOW | | LOW | | MEDIUM | | LOW | | LOW | | MEDIUM | |
| 10.3389/fnins.2023.1234286 | | | | Borelli et al., 2023 | | | | LOW | | LOW | | MEDIUM | | LOW | | LOW | | MEDIUM | |
| 10.1016/j.neuroimage.2007.02.001 | | | | Moulton et al., 2007 | | | | LOW | | MEDIUM | | MEDIUM | | LOW | | MEDIUM | | MEDIUM | |
| 10.7554/eLife.97793 | | | | Wittkamp et al., 2024 | | | | LOW | | LOW | | MEDIUM | | LOW | | LOW | | MEDIUM | |
| 10.1016/j.pain.2005.01.005 | | | | Zambreanu et al., 2005 | | | | LOW | | LOW | | MEDIUM | | LOW | | MEDIUM | | MEDIUM | |
| 10.1097/j.pain.0000000000002814 | | | | Peng et al., 2023 | | | | LOW | | LOW | | LOW | | LOW | | LOW | | LOW | |
| 10.1002/j.1532-2149.2011.00027.x | | | | Benson et al., 2012 | | | | LOW | | MEDIUM | | MEDIUM | | LOW | | LOW | | MEDIUM | |
| 10.1523/jneurosci.0006-12.2012 | | | | Moulton et al., 2012 | | | | LOW | | MEDIUM | | MEDIUM | | LOW | | LOW | | MEDIUM | |
| 10.1002/hbm.22035 | | | | Seifert et al., 2013 | | | | LOW | | MEDIUM | | MEDIUM | | LOW | | MEDIUM | | MEDIUM | |
| 10.1016/j.neures.2011.01.015 | | | | Uematsu et al., 2011 | | | | LOW | | MEDIUM | | MEDIUM | | LOW | | LOW | | MEDIUM | |
| 10.1016/j.neuroimage.2019.01.039 | | | | Su et al., 2019 | | | | LOW | | LOW | | MEDIUM | | LOW | | LOW | | MEDIUM | |
| 10.1523/jneurosci.1954-15.2016 | | | | Bräscher et al., 2016 | | | | LOW | | MEDIUM | | MEDIUM | | LOW | | LOW | | MEDIUM | |
| 10.1016/j.pain.2008.10.027 | | | | Veldhuijzen et al., 2009 | | | | LOW | | LOW | | MEDIUM | | LOW | | LOW | | MEDIUM | |
| 10.1016/j.pain.2004.02.033 | | | | Valet et al., 2004 | | | | LOW | | MEDIUM | | MEDIUM | | LOW | | LOW | | MEDIUM | |
| 10.1016/j.pain.2013.06.041 | | | | Tseng et al., 2013 | | | | LOW | | MEDIUM | | MEDIUM | | LOW | | LOW | | MEDIUM | |
| 10.1002/hbm.24824 | | | | Letzen et al., 2020 | | | | LOW | | LOW | | MEDIUM | | LOW | | LOW | | MEDIUM | |
| 10.1073/pnas.1511269112 | | | | Rütgen et al., 2015 | | | | LOW | | LOW | | LOW | | LOW | | MEDIUM | | MEDIUM | |
| ***chronic pain*** | | |  | | | | |  | |  | |  | |  | |  | |  | |
| 10.1371/journal.pone.0156545 | | | | Hubbard et al., 2016 | | | | LOW | | HIGH | | MEDIUM | | LOW | | LOW | | MEDIUM | |
| 10.1177/0022034521994089 | | | | Lim et al., 2021 | | | | LOW | | HIGH | | MEDIUM | | MEDIUM | | LOW | | HIGH | |
| 10.1371/journal.pone.0221023 | | | | Ihara et al., 2019 | | | | LOW | | HIGH | | MEDIUM | | LOW | | LOW | | MEDIUM | |
| 10.1002/art.41220 | | | | Hubbard et al., 2020 | | | | LOW | | HIGH | | MEDIUM | | LOW | | LOW | | HIGH | |
| 10.2147/jpr.S327945 | | | | Zhu et al., 2021 | | | | LOW | | HIGH | | MEDIUM | | MEDIUM | | LOW | | HIGH | |
| 10.1186/s10194-021-01381-w | | | | Mungoven et al., 2022 | | | | LOW | | HIGH | | MEDIUM | | MEDIUM | | LOW | | MEDIUM | |
| 10.1111/head.14360 | | | | Szabo et al., 2022 | | | | LOW | | HIGH | | MEDIUM | | LOW | | LOW | | HIGH | |
| 10.1016/j.ejpn.2022.10.004 | | | | Szabo et al., 2022 | | | | LOW | | HIGH | | MEDIUM | | LOW | | LOW | | HIGH | |
| 10.1097/j.pain.0000000000000951 | | | | Wang et al., 2017 | | | | LOW | | HIGH | | MEDIUM | | MEDIUM | | LOW | | HIGH | |
| 10.1097/psy.0000000000000655 | | | | Bhatt et al., 2019 | | | | LOW | | HIGH | | MEDIUM | | MEDIUM | | LOW | | MEDIUM | |
| 10.1016/j.bbi.2019.06.024 | | | | Sandstrom et al., 2019 | | | | LOW | | HIGH | | MEDIUM | | LOW | | LOW | | MEDIUM | |
| 10.1097/wnr.0000000000000529 | | | | Wang et al., 2016 | | | | LOW | | HIGH | | MEDIUM | | LOW | | LOW | | HIGH | |
| 10.1007/s00234-020-02582-x | | | | Dai et al., 2021 | | | | LOW | | HIGH | | MEDIUM | | MEDIUM | | LOW | | MEDIUM | |
| 10.1186/s10194-016-0610-4 | | | | Wang et al., 2016 | | | | LOW | | HIGH | | MEDIUM | | MEDIUM | | LOW | | MEDIUM | |
| 10.3389/fpain.2022.918766 | | | | Verriotis et al., 2022 | | | | LOW | | HIGH | | MEDIUM | | MEDIUM | | LOW | | HIGH | |
| 10.1007/s00540-017-2343-1 | | | | Matsuo et al., 2017 | | | | LOW | | HIGH | | MEDIUM | | MEDIUM | | LOW | | MEDIUM | |
| 10.1093/cercor/bhp205 | | | | Gustin et al., 2010 | | | | LOW | | HIGH | | MEDIUM | | LOW | | LOW | | MEDIUM | |
| 10.1002/j.1532-2149.2011.00058.x | | | | Burgmer et al., 2012 | | | | LOW | | HIGH | | MEDIUM | | LOW | | LOW | | MEDIUM | |
| 10.1016/j.pain.2013.11.008 | | | | Youssef et al., 2014 | | | | LOW | | HIGH | | MEDIUM | | MEDIUM | | LOW | | HIGH | |
| 10.1186/s10194-017-0747-9 | | | | Chen et al., 2017 | | | | LOW | | HIGH | | MEDIUM | | LOW | | LOW | | MEDIUM | |
| 10.1016/j.jpain.2023.05.006 | | | | Sandstrom et al., 2023 | | | | LOW | | HIGH | | MEDIUM | | LOW | | LOW | | MEDIUM | |
| 10.3389/fnhum.2016.00489 | | | | Mathur et al., 2016 | | | | LOW | | HIGH | | MEDIUM | | MEDIUM | | LOW | | HIGH | |
| 10.1097/j.pain.0000000000001907 | | | | Sandstrom et al., 2020 | | | | LOW | | HIGH | | MEDIUM | | LOW | | LOW | | MEDIUM | |
| 10.1016/j.nicl.2023.103355 | | | | Mosch et al., 2023 | | | | LOW | | HIGH | | MEDIUM | | LOW | | LOW | | HIGH | |
| 10.1093/brain/awh098 | | | | Gracely et al., 2004 | | | | LOW | | HIGH | | MEDIUM | | LOW | | LOW | | HIGH | |
| 10.1002/hbm.22757 | | | | Ceko et al., 2015 | | | | LOW | | HIGH | | MEDIUM | | MEDIUM | | LOW | | HIGH | |
| 10.1002/hbm.25401 | | | | Huynh et al., 2021 | | | | LOW | | HIGH | | MEDIUM | | LOW | | LOW | | HIGH | |
| 10.1016/j.neulet.2014.08.027 | | | | Hiramatsu et al., 2014 | | | | LOW | | HIGH | | MEDIUM | | MEDIUM | | LOW | | HIGH | |
| https://doi.org/10.1111/head.14900 | | | | Zhao et al., 2025 | | | | LOW | | HIGH | | MEDIUM | | LOW | | LOW | | MEDIUM | |
| 10.1523/eneuro.0522-23.2024 | | | | Meylakh et al., 2024 | | | | LOW | | HIGH | | MEDIUM | | MEDIUM | | LOW | | HIGH | |
| 10.2147/jpr.S500924 | | | | Gao et al., 2025 | | | | LOW | | HIGH | | MEDIUM | | LOW | | LOW | | MEDIUM | |
| 10.21037/qims-23-1687 | | | | Jin et al., 2024 | | | | LOW | | HIGH | | MEDIUM | | MEDIUM | | LOW | | HIGH | |
| 10.1007/s40122-022-00365-1 | | | | Cao et al., 2022 | | | | LOW | | HIGH | | MEDIUM | | LOW | | LOW | | HIGH | |
| 10.1111/pme.12267 | | | | Schwedt et al., 2014 | | | | LOW | | HIGH | | MEDIUM | | MEDIUM | | LOW | | HIGH | |
| 10.23736/s1973-9087.24.08087-0 | | | | Zhang et al., 2024 | | | | LOW | | LOW | | LOW | | MEDIUM | | LOW | | HIGH | |
| 10.1016/j.neuroimage.2008.09.008 | | | | Burgmer et al., 2009 | | | | LOW | | HIGH | | MEDIUM | | MEDIUM | | LOW | | HIGH | |
| 10.1016/j.clinph.2014.10.004 | | | | Ma et al., 2015 | | | | LOW | | HIGH | | MEDIUM | | MEDIUM | | LOW | | HIGH | |
| 10.1002/ana.22537 | | | | Mainero et al., 2011 | | | | LOW | | HIGH | | MEDIUM | | MEDIUM | | LOW | | HIGH | |
| 10.1016/j.brainres.2021.147608 | | | | Tang et al., 2021 | | | | LOW | | HIGH | | MEDIUM | | MEDIUM | | LOW | | MEDIUM | |
| 10.1371/journal.pone.0004542 | | | | Cauda et al., 2009 | | | | LOW | | HIGH | | MEDIUM | | MEDIUM | | LOW | | HIGH | |
| 10.1002/hbm.25038 | | | | Mao et al., 2020 | | | | LOW | | HIGH | | MEDIUM | | MEDIUM | | LOW | | HIGH | |
| 10.1002/hbm.23696 | | | | Tsai et al., 2018 | | | | LOW | | HIGH | | MEDIUM | | MEDIUM | | LOW | | HIGH | |
| 10.1097/j.pain.0000000000002933 | | | | Tong et al., 2023 | | | | LOW | | HIGH | | MEDIUM | | MEDIUM | | LOW | | HIGH | |
| 10.1111/j.1365-2982.2005.00720.x | | | | Andresen et al., 2005 | | | | LOW | | HIGH | | MEDIUM | | MEDIUM | | LOW | | HIGH | |
| 10.1002/acr.23601 | | | | Lee et al., 2019 | | | | LOW | | HIGH | | MEDIUM | | MEDIUM | | LOW | | HIGH | |
| 10.1515/sjpain-2018-0341 | | | | Kornelsen et al., 2013 | | | | LOW | | HIGH | | MEDIUM | | MEDIUM | | LOW | | MEDIUM | |
| 10.1186/s10194-020-01210-6 | | | | Lim et al., 2021 | | | | LOW | | HIGH | | MEDIUM | | MEDIUM | | LOW | | HIGH | |
| 10.3389/fnhum.2018.00107 | | | | Zhang et al., 2018 | | | | LOW | | HIGH | | MEDIUM | | MEDIUM | | LOW | | HIGH | |
| 10.1097/j.pain.0000000000000633 | | | | Schrepf et al., 2016 | | | | MEDIUM | | HIGH | | MEDIUM | | MEDIUM | | LOW | | HIGH | |
| 10.1177/0333102414526069 | | | | Schwedt et al., 2014 | | | | LOW | | HIGH | | MEDIUM | | MEDIUM | | LOW | | HIGH | |
| 10.1097/j.pain.0000000000001366 | | | | Roy et al., 2018 | | | | LOW | | HIGH | | MEDIUM | | MEDIUM | | LOW | | HIGH | |
| ***placebo analgesia*** | | | | |  | | |  | |  | |  | |  | |  | |  | |
| 10.3389/fnbeh.2021.696577 | | | | Shi et al., 2021 | | | | LOW | | MEDIUM | | HIGH | | MEDIUM | | LOW | | MEDIUM | |
| 10.1016/j.pain.2012.08.008 | | | | Hashmi et al., 2012 | | | | LOW | | MEDIUM | | MEDIUM | | MEDIUM | | LOW | | MEDIUM | |
| 10.1016/j.jpain.2015.05.001 | | | | Sevel et al., 2015 | | | | LOW | | MEDIUM | | MEDIUM | | MEDIUM | | LOW | | MEDIUM | |
| 10.1093/cercor/bhad247 | | | | Crawford et al., 2023 | | | | LOW | | MEDIUM | | MEDIUM | | LOW | | MEDIUM | | MEDIUM | |
| 10.1016/j.pain.2013.03.031 | | | | Huber et al., 2013 | | | | LOW | | MEDIUM | | MEDIUM | | MEDIUM | | LOW | | MEDIUM | |
| 10.1073/pnas.2101273118 | | | | Tu et al., 2021 | | | | LOW | | LOW | | LOW | | LOW | | MEDIUM | | MEDIUM | |
| 10.1016/j.pain.2011.10.036 | | | | Elsenbruch et al., 2012 | | | | LOW | | LOW | | MEDIUM | | LOW | | LOW | | MEDIUM | |
| 10.1155/2020/8820443 | | | | Shi et al., 2020 | | | | LOW | | MEDIUM | | MEDIUM | | LOW | | LOW | | MEDIUM | |
| 10.1016/j.neuroimage.2015.01.056 | | | | Sevel et al., 2015 | | | | LOW | | MEDIUM | | MEDIUM | | MEDIUM | | LOW | | MEDIUM | |
| 10.1016/j.pain.2009.04.003 | | | | Watson et al., 2009 | | | | LOW | | MEDIUM | | MEDIUM | | MEDIUM | | MEDIUM | | MEDIUM | |
| 10.1016/j.nicl.2014.09.007 | | | | Schmidt-Wilcke et al., 2014 | | | | LOW | | LOW | | MEDIUM | | MEDIUM | | LOW | | MEDIUM | |
| 10.1016/j.neuroimage.2019.116510 | | | | Schenk & Colloca., 2020 | | | | LOW | | LOW | | MEDIUM | | LOW | | LOW | | MEDIUM | |
| 10.1093/scan/nsx033 | | | | Van Der Meulen et al., 2017 | | | | LOW | | MEDIUM | | MEDIUM | | LOW | | LOW | | HIGH | |
| 10.1111/nmo.12454 | | | | Theysohn et al., 2014 | | | | LOW | | LOW | | HIGH | | MEDIUM | | LOW | | MEDIUM | |
| 10.1038/s41598-024-77693-z | | | | Wolf et al., 2024 | | | | LOW | | MEDIUM | | MEDIUM | | MEDIUM | | LOW | | HIGH | |
| 10.1016/j.neuron.2009.07.014 | | | | Eippert et al., 2009 | | | | LOW | | LOW | | LOW | | MEDIUM | | MEDIUM | | LOW | |
| 10.1523/jneurosci.3556-05.2006 | | | | Kong et al., 2006 | | | | LOW | | MEDIUM | | MEDIUM | | MEDIUM | | MEDIUM | | HIGH | |
| 10.1016/j.neuroimage.2012.11.029 | | | | Geuter et al., 2013 | | | | LOW | | LOW | | MEDIUM | | MEDIUM | | MEDIUM | | HIGH | |
| 10.1523/jneurosci.2542-15.2015 | | | | Zeidan et al., 2015 | | | | LOW | | LOW | | MEDIUM | | LOW | | LOW | | MEDIUM | |
| 10.1016/j.pain.2010.09.021 | | | | Lui et al., 2010 | | | | LOW | | MEDIUM | | MEDIUM | | MEDIUM | | LOW | | HIGH | |
| 10.1038/s41598-017-18870-1 | | | | Makary et al., 2018 | | | | LOW | | MEDIUM | | MEDIUM | | LOW | | MEDIUM | | HIGH | |
| 10.1111/pme.12783 | | | | Fehse et al., 2015 | | | | LOW | | MEDIUM | | MEDIUM | | LOW | | LOW | | HIGH | |
| ***conditioned pain modulation*** | | | | | |  | |  | |  | |  | |  | |  | |  | |
| 10.1097/pr9.0000000000000999 | | | | Harrison et al., 2022 | | | | LOW | | MEDIUM | | MEDIUM | | LOW | | LOW | | HIGH | |
| 10.1002/hbm.23199 | | | | Youssef et al., 2016 | | | | LOW | | MEDIUM | | LOW | | MEDIUM | | MEDIUM | | MEDIUM | |
| 10.1016/j.brainres.2011.11.056 | | | | Moont et al., 2012 | | | | LOW | | MEDIUM | | HIGH | | MEDIUM | | LOW | | HIGH | |
| 10.1016/j.pain.2006.06.017 | | | | Song et al., 2006 | | | | LOW | | MEDIUM | | MEDIUM | | LOW | | LOW | | MEDIUM | |
| 10.1016/j.neuroimage.2021.118742 | | | | Huynh et al., 2022 | | | | LOW | | LOW | | MEDIUM | | MEDIUM | | LOW | | HIGH | |
| 10.1016/j.pain.2014.07.008 | | | | Nahman-Averbuch et al., 2014 | | | | LOW | | MEDIUM | | MEDIUM | | MEDIUM | | LOW | | HIGH | |
| ***Offset analgesia*** | |  | | | | | |  | |  | |  | |  | |  | |  | |
| 10.1177/1744806918767512 | | | | Zhang et al., 2018 | | | | LOW | | MEDIUM | | MEDIUM | | MEDIUM | | LOW | | HIGH | |
| 10.1016/j.pain.2014.07.008 | | | | Nahman-Averbuch et al., 2014 | | | | LOW | | MEDIUM | | MEDIUM | | MEDIUM | | LOW | | HIGH | |
| ***miscellaneous pain modulation*** | | | | | | |  | |  | |  | |  | |  | |  | |  |
| 10.1097/j.pain.0000000000002814 | | | | Peng et al., 2023  (non-invasive brain stimulation) | | | | LOW | | LOW | | LOW | | MEDIUM | | LOW | | LOW | |
| 10.1523/jneurosci.2568-06.2006 | | | | Weich et al., 2006  (expectancy pain modulation) | | | | LOW | | LOW | | MEDIUM | | LOW | | LOW | | HIGH | |
| 10.1002/ejp.696 | | | | Choi et al., 2016  (TENS analgesia) | | | | LOW | | LOW | | MEDIUM | | LOW | | LOW | | HIGH | |
| 10.1097/j.pain.0000000000001599 | | | | Lopez-Sola et al., 2019 (emotional pain modulation) | | | | LOW | | MEDIUM | | HIGH | | LOW | | LOW | | HIGH | |
| 10.1371/journal.pone.0110654 | | | | Wiech et al., 2014 (emotional pain modulation) | | | | LOW | | MEDIUM | | MEDIUM | | LOW | | LOW | | HIGH | |
| 10.1093/scan/nsad018 | | | | Silvestrini et al., 2023 (attentional analgesia) | | | | MEDIUM | | MEDIUM | | HIGH | | HIGH | | MEDIUM | | MEDIUM | |
| 10.3389/fnins.2020.00006 | | | | Henderson et al., 2020 (expectancy pain modulation) | | | | LOW | | MEDIUM | | MEDIUM | | MEDIUM | | LOW | | HIGH | |
| 10.1038/s41598-019-51990-4 | | | | Pando-Naude et al., 2019 (music analgesia) | | | | LOW | | LOW | | MEDIUM | | LOW | | LOW | | HIGH | |
| 10.1002/j.1532-2149.2011.00039.x | | | | Godinho et al., 2012 (emotional pain modulation) | | | | LOW | | MEDIUM | | MEDIUM | | LOW | | LOW | | HIGH | |
| 10.1016/j.jpain.2014.07.006 | | | | Dobek et al., 2014 (music analgesia) | | | | LOW | | LOW | | HIGH | | LOW | | LOW | | HIGH | |
| 10.1016/j.neuroimage.2009.05.031 | | | | Vanhaudenhuyse et al., 2009 (hypnosis analgesia) | | | | LOW | | LOW | | MEDIUM | | LOW | | LOW | | HIGH | |
| 10.1002/hbm.20716 | | | | Raij et al., 2009 (expectancy pain modulation) | | | | LOW | | MEDIUM | | MEDIUM | | LOW | | MEDIUM | | LOW | |
| 10.1186/1744-8069-7-45 | | | | Shukla et al., 2011 (acupuncture analgesia) | | | | LOW | | LOW | | HIGH | | LOW | | LOW | | HIGH | |
| 10.1089/brain.2016.0440 | | | | Sankarasubramanian et al., 2017 (non-invasive brain stimulation) | | | | LOW | | LOW | | LOW | | MEDIUM | | LOW | | MEDIUM | |
| 10.1007/s11682-018-9875-3 | | | | Kong et al., 2019 (exercise analgesia) | | | | MEDIUM | | HIGH | | MEDIUM | | LOW | | MEDIUM | | HIGH | |
| 10.1111/j.1365-2982.2007.00908.x | | | | Dunckley et al., 2007 (attentional analgesia) | | | | LOW | | LOW | | HIGH | | MEDIUM | | MEDIUM | | HIGH | |
| 10.1155/2015/210120 | | | | Shi et al., 2015 (acupuncture analgesia) | | | | LOW | | MEDIUM | | MEDIUM | | MEDIUM | | LOW | | HIGH | |
| 10.1093/pm/pnaa178 | | | | Berry et al., 2020 (emotional pain modulation) | | | | LOW | | HIGH | | MEDIUM | | LOW | | MEDIUM | | LOW | |
| 10.1016/j.ejpain.2011.07.010 | | | | Mohr et al., 2012 (expectancy pain modulation) | | | | LOW | | MEDIUM | | MEDIUM | | LOW | | MEDIUM | | LOW | |
| 10.1016/j.nicl.2018.01.021 | | | | Kong et al., 2018 (expectancy pain modulation) | | | | LOW | | MEDIUM | | MEDIUM | | LOW | | MEDIUM | | MEDIUM | |
| 10.3390/brainsci6010008 | | | | Ellingson et al., 2016 (exercise analgesia) | | | | LOW | | LOW | | HIGH | | LOW | | LOW | | HIGH | |
| 10.1523/jneurosci.2542-15.2015 | | | | Zeidan et al., 2015 (emotional pain modulation) | | | | LOW | | MEDIUM | | LOW | | LOW | | LOW | | MEDIUM | |
| 10.1038/npp.2013.13 | | | | Taylor et al., 2013 (non-invasive brain stimulation) | | | | MEDIUM | | LOW | | MEDIUM | | LOW | | MEDIUM | | HIGH | |
| 10.1016/j.neuroimage.2020.117548 | | | | Oliva et al., 2021 (attentional analgesia) | | | | LOW | | LOW | | MEDIUM | | LOW | | MEDIUM | | MEDIUM | |
| 10.1186/s10194-018-0924-5 | | | | Naegel et al., 2018 (non-invasive brain stimulation) | | | | LOW | | LOW | | LOW | | LOW | | MEDIUM | | HIGH | |
| 10.1038/s41598-022-21557-x | | | | Argaman et al., 2022 (non-invasive brain stimulation) | | | | LOW | | MEDIUM | | MEDIUM | | LOW | | LOW | | MEDIUM | |
| 10.1523/jneurosci.2123-21.2022 | | | | Zhang et al., 2022 (emotional pain modulation) | | | | LOW | | LOW | | MEDIUM | | LOW | | LOW | | MEDIUM | |
| 10.1515/sjpain-2018-0341 | | | | Kornelsen et al., 2019 (emotional pain modulation) | | | | LOW | | MEDIUM | | MEDIUM | | LOW | | LOW | | MEDIUM | |
| 10.1371/journal.pone.0013309 | | | | Younger et al., 2010 (emotional pain modulation) | | | | MEDIUM | | MEDIUM | | MEDIUM | | MEDIUM | | LOW | | MEDIUM | |

**References**

Andresen, V., Bach, D. R., Poellinger, A., Tsrouya, C., Stroh, A., Foerschler, A., . . . Mönnikes, H. (2005). Brain activation responses to subliminal or supraliminal rectal stimuli and to auditory stimuli in irritable bowel syndrome. *Neurogastroenterol Motil, 17*(6), 827-837. doi:10.1111/j.1365-2982.2005.00720.x

Argaman, Y., Granovsky, Y., Sprecher, E., Sinai, A., Yarnitsky, D., & Weissman-Fogel, I. (2022). Resting-state functional connectivity predicts motor cortex stimulation-dependent pain relief in fibromyalgia syndrome patients. *Sci Rep, 12*(1), 17135. doi:10.1038/s41598-022-21557-x

Atlas, L. Y., Lindquist, M. A., Bolger, N., & Wager, T. D. (2014). Brain mediators of the effects of noxious heat on pain. *Pain, 155*(8), 1632-1648. doi:10.1016/j.pain.2014.05.015

Benson, S., Kotsis, V., Rosenberger, C., Bingel, U., Forsting, M., Schedlowski, M., . . . Elsenbruch, S. (2012). Behavioural and neural correlates of visceral pain sensitivity in healthy men and women: does sex matter? *Eur J Pain, 16*(3), 349-358. doi:10.1002/j.1532-2149.2011.00027.x

Berry, M. P., Lutz, J., Schuman-Olivier, Z., Germer, C., Pollak, S., Edwards, R. R., . . . Napadow, V. (2020). Brief Self-Compassion Training Alters Neural Responses to Evoked Pain for Chronic Low Back Pain: A Pilot Study. *Pain Med, 21*(10), 2172-2185. doi:10.1093/pm/pnaa178

Bhatt, R. R., Gupta, A., Labus, J. S., Zeltzer, L. K., Tsao, J. C., Shulman, R. J., & Tillisch, K. (2019). Altered Brain Structure and Functional Connectivity and Its Relation to Pain Perception in Girls With Irritable Bowel Syndrome. *Psychosom Med, 81*(2), 146-154. doi:10.1097/psy.0000000000000655

Borelli, E., Benuzzi, F., Ballotta, D., Bandieri, E., Luppi, M., Cacciari, C., . . . Lui, F. (2023). Words hurt: common and distinct neural substrates underlying nociceptive and semantic pain. *Front Neurosci, 17*, 1234286. doi:10.3389/fnins.2023.1234286

Bornhövd, K., Quante, M., Glauche, V., Bromm, B., Weiller, C., & Büchel, C. (2002). Painful stimuli evoke different stimulus-response functions in the amygdala, prefrontal, insula and somatosensory cortex: a single-trial fMRI study. *Brain, 125*(Pt 6), 1326-1336. doi:10.1093/brain/awf137

Bräscher, A. K., Becker, S., Hoeppli, M. E., & Schweinhardt, P. (2016). Different Brain Circuitries Mediating Controllable and Uncontrollable Pain. *J Neurosci, 36*(18), 5013-5025. doi:10.1523/jneurosci.1954-15.2016

Burgmer, M., Pfleiderer, B., Maihöfner, C., Gaubitz, M., Wessolleck, E., Heuft, G., & Pogatzki-Zahn, E. (2012). Cerebral mechanisms of experimental hyperalgesia in fibromyalgia. *Eur J Pain, 16*(5), 636-647. doi:10.1002/j.1532-2149.2011.00058.x

Burgmer, M., Pogatzki-Zahn, E., Gaubitz, M., Wessoleck, E., Heuft, G., & Pfleiderer, B. (2009). Altered brain activity during pain processing in fibromyalgia. *Neuroimage, 44*(2), 502-508. doi:10.1016/j.neuroimage.2008.09.008

Cao, Z. M., Chen, Y. C., Liu, G. Y., Wang, X., Shi, A. Q., Xu, L. F., . . . Wang, J. (2022). Abnormalities of Thalamic Functional Connectivity in Patients with Migraine: A Resting-State fMRI Study. *Pain Ther, 11*(2), 561-574. doi:10.1007/s40122-022-00365-1

Cauda, F., Sacco, K., Duca, S., Cocito, D., D'Agata, F., Geminiani, G. C., & Canavero, S. (2009). Altered resting state in diabetic neuropathic pain. *PLoS One, 4*(2), e4542. doi:10.1371/journal.pone.0004542

Čeko, M., Shir, Y., Ouellet, J. A., Ware, M. A., Stone, L. S., & Seminowicz, D. A. (2015). Partial recovery of abnormal insula and dorsolateral prefrontal connectivity to cognitive networks in chronic low back pain after treatment. *Hum Brain Mapp, 36*(6), 2075-2092. doi:10.1002/hbm.22757

Chen, Z., Chen, X., Liu, M., Liu, S., Ma, L., & Yu, S. (2017). Disrupted functional connectivity of periaqueductal gray subregions in episodic migraine. *J Headache Pain, 18*(1), 36. doi:10.1186/s10194-017-0747-9

Choi, J. C., Kim, J., Kang, E., Lee, J. M., Cha, J., Kim, Y. J., . . . Yi, D. J. (2016). Brain mechanisms of pain relief by transcutaneous electrical nerve stimulation: A functional magnetic resonance imaging study. *Eur J Pain, 20*(1), 92-105. doi:10.1002/ejp.696

Crawford, L., Mills, E., Meylakh, N., Macey, P. M., Macefield, V. G., & Henderson, L. A. (2023). Brain activity changes associated with pain perception variability. *Cereb Cortex, 33*(7), 4145-4155. doi:10.1093/cercor/bhac332

Crawford, L. S., Mills, E. P., Peek, A., Macefield, V. G., Keay, K. A., & Henderson, L. A. (2023). Function and biochemistry of the dorsolateral prefrontal cortex during placebo analgesia: how the certainty of prior experiences shapes endogenous pain relief. *Cerebral Cortex, 33*(17), 9822-9834. doi:10.1093/cercor/bhad247

Dai, L., Yu, Y., Zhao, H., Zhang, X., Su, Y., Wang, X., . . . Ke, J. (2021). Altered local and distant functional connectivity density in chronic migraine: a resting-state functional MRI study. *Neuroradiology, 63*(4), 555-562. doi:10.1007/s00234-020-02582-x

Dobek, C. E., Beynon, M. E., Bosma, R. L., & Stroman, P. W. (2014). Music modulation of pain perception and pain-related activity in the brain, brain stem, and spinal cord: a functional magnetic resonance imaging study. *J Pain, 15*(10), 1057-1068. doi:10.1016/j.jpain.2014.07.006

Dunckley, P., Aziz, Q., Wise, R. G., Brooks, J., Tracey, I., & Chang, L. (2007). Attentional modulation of visceral and somatic pain. *Neurogastroenterol Motil, 19*(7), 569-577. doi:10.1111/j.1365-2982.2007.00908.x

Eippert, F., Bingel, U., Schoell, E. D., Yacubian, J., Klinger, R., Lorenz, J., & Büchel, C. (2009). Activation of the opioidergic descending pain control system underlies placebo analgesia. *Neuron, 63*(4), 533-543. doi:10.1016/j.neuron.2009.07.014

Ellingson, L. D., Stegner, A. J., Schwabacher, I. J., Koltyn, K. F., & Cook, D. B. (2016). Exercise Strengthens Central Nervous System Modulation of Pain in Fibromyalgia. *Brain Sci, 6*(1). doi:10.3390/brainsci6010008

Elsenbruch, S., Kotsis, V., Benson, S., Rosenberger, C., Reidick, D., Schedlowski, M., . . . Gizewski, E. R. (2012). Neural mechanisms mediating the effects of expectation in visceral placebo analgesia: an fMRI study in healthy placebo responders and nonresponders. *Pain, 153*(2), 382-390. doi:10.1016/j.pain.2011.10.036

Fehse, K., Maikowski, L., Simmank, F., Gutyrchik, E., & Meissner, K. (2015). Placebo Responses to Original vs. Generic ASA Brands During Exposure to Noxious Heat: A Pilot fMRI Study of Neurofunctional Correlates. *Pain Med, 16*(10), 1967-1974. doi:10.1111/pme.12783

Forkmann, K., Grashorn, W., Schmidt, K., Fründt, O., Buhmann, C., & Bingel, U. (2017). Altered neural responses to heat pain in drug-naive patients with Parkinson disease. *Pain, 158*(8), 1408-1416. doi:10.1097/j.pain.0000000000000923

Freund, W., Klug, R., Weber, F., Stuber, G., Schmitz, B., & Wunderlich, A. P. (2009). Perception and suppression of thermally induced pain: a fMRI study. *Somatosens Mot Res, 26*(1), 1-10. doi:10.1080/08990220902738243

Gao, Z., Cui, M. J., Wang, H. J., Zhang, J., Xu, C., & Ji, L. X. (2025). Investigating Brain Structure and Functional Alterations in the Transition from Acute to Chronic Neck Pain: A Resting-State fMRI Study. *J Pain Res, 18*, 579-587. doi:10.2147/jpr.S500924

Geuter, S., Eippert, F., Hindi Attar, C., & Büchel, C. (2013). Cortical and subcortical responses to high and low effective placebo treatments. *Neuroimage, 67*, 227-236. doi:10.1016/j.neuroimage.2012.11.029

Godinho, F., Faillenot, I., Perchet, C., Frot, M., Magnin, M., & Garcia-Larrea, L. (2012). How the pain of others enhances our pain: searching the cerebral correlates of 'compassional hyperalgesia'. *Eur J Pain, 16*(5), 748-759. doi:10.1002/j.1532-2149.2011.00039.x

González-Roldán, A. M., Terrasa, J. L., Sitges, C., van der Meulen, M., Anton, F., & Montoya, P. (2020). Age-Related Changes in Pain Perception Are Associated With Altered Functional Connectivity During Resting State. *Front Aging Neurosci, 12*, 116. doi:10.3389/fnagi.2020.00116

Gracely, R. H., Geisser, M. E., Giesecke, T., Grant, M. A., Petzke, F., Williams, D. A., & Clauw, D. J. (2004). Pain catastrophizing and neural responses to pain among persons with fibromyalgia. *Brain, 127*(Pt 4), 835-843. doi:10.1093/brain/awh098

Gustin, S. M., Wrigley, P. J., Henderson, L. A., & Siddall, P. J. (2010). Brain circuitry underlying pain in response to imagined movement in people with spinal cord injury. *Pain, 148*(3), 438-445. doi:10.1016/j.pain.2009.12.001

Harrison, R., Gandhi, W., van Reekum, C. M., & Salomons, T. V. (2022). Conditioned pain modulation is associated with heightened connectivity between the periaqueductal grey and cortical regions. *Pain Rep, 7*(3), e999. doi:10.1097/pr9.0000000000000999

Hashmi, J. A., Baria, A. T., Baliki, M. N., Huang, L., Schnitzer, T. J., & Apkarian, V. A. (2012). Brain networks predicting placebo analgesia in a clinical trial for chronic back pain. *Pain, 153*(12), 2393-2402. doi:10.1016/j.pain.2012.08.008

Henderson, L. A., Di Pietro, F., Youssef, A. M., Lee, S., Tam, S., Akhter, R., . . . Macey, P. M. (2020). Effect of Expectation on Pain Processing: A Psychophysics and Functional MRI Analysis. *Front Neurosci, 14*, 6. doi:10.3389/fnins.2020.00006

Hiramatsu, T., Nakanishi, K., Yoshimura, S., Yoshino, A., Adachi, N., Okamoto, Y., . . . Ochi, M. (2014). The dorsolateral prefrontal network is involved in pain perception in knee osteoarthritis patients. *Neurosci Lett, 581*, 109-114. doi:10.1016/j.neulet.2014.08.027

Hubbard, C. S., Becerra, L., Heinz, N., Ludwick, A., Rasooly, T., Wu, R., . . . Nurko, S. (2016). Abdominal Pain, the Adolescent and Altered Brain Structure and Function. *PLoS One, 11*(5), e0156545. doi:10.1371/journal.pone.0156545

Hubbard, C. S., Lazaridou, A., Cahalan, C. M., Kim, J., Edwards, R. R., Napadow, V., & Loggia, M. L. (2020). Aberrant Salience? Brain Hyperactivation in Response to Pain Onset and Offset in Fibromyalgia. *Arthritis Rheumatol, 72*(7), 1203-1213. doi:10.1002/art.41220

Huber, A., Lui, F., & Porro, C. A. (2013). Hypnotic susceptibility modulates brain activity related to experimental placebo analgesia. *Pain, 154*(9), 1509-1518. doi:10.1016/j.pain.2013.03.031

Huynh, V., Lütolf, R., Rosner, J., Luechinger, R., Curt, A., Kollias, S., . . . Michels, L. (2021). Supraspinal nociceptive networks in neuropathic pain after spinal cord injury. *Hum Brain Mapp, 42*(12), 3733-3749. doi:10.1002/hbm.25401

Huynh, V., Lütolf, R., Rosner, J., Luechinger, R., Curt, A., Kollias, S., . . . Hubli, M. (2022). Descending pain modulatory efficiency in healthy subjects is related to structure and resting connectivity of brain regions. *Neuroimage, 247*, 118742. doi:10.1016/j.neuroimage.2021.118742

Ihara, N., Wakaizumi, K., Nishimura, D., Kato, J., Yamada, T., Suzuki, T., . . . Morisaki, H. (2019). Aberrant resting-state functional connectivity of the dorsolateral prefrontal cortex to the anterior insula and its association with fear avoidance belief in chronic neck pain patients. *PLoS One, 14*(8), e0221023. doi:10.1371/journal.pone.0221023

Jin, P., Wang, F., Zeng, F., Yu, J., Cui, F., Yang, B., & Zhang, L. (2024). Revealing the mechanism of central pain hypersensitivity in primary dysmenorrhea: evidence from neuroimaging. *Quant Imaging Med Surg, 14*(4), 3075-3085. doi:10.21037/qims-23-1687

Kong, J., Gollub, R. L., Rosman, I. S., Webb, J. M., Vangel, M. G., Kirsch, I., & Kaptchuk, T. J. (2006). Brain activity associated with expectancy-enhanced placebo analgesia as measured by functional magnetic resonance imaging. *J Neurosci, 26*(2), 381-388. doi:10.1523/jneurosci.3556-05.2006

Kong, J., Wang, Z., Leiser, J., Minicucci, D., Edwards, R., Kirsch, I., . . . Gollub, R. L. (2018). Enhancing treatment of osteoarthritis knee pain by boosting expectancy: A functional neuroimaging study. *Neuroimage Clin, 18*, 325-334. doi:10.1016/j.nicl.2018.01.021

Kong, J., Wolcott, E., Wang, Z., Jorgenson, K., Harvey, W. F., Tao, J., . . . Wang, C. (2019). Altered resting state functional connectivity of the cognitive control network in fibromyalgia and the modulation effect of mind-body intervention. *Brain Imaging Behav, 13*(2), 482-492. doi:10.1007/s11682-018-9875-3

Kornelsen, J., McIver, T. A., & Stroman, P. W. (2019). Unique brain regions involved in positive versus negative emotional modulation of pain. *Scand J Pain, 19*(3), 583-596. doi:10.1515/sjpain-2018-0341

Kornelsen, J., Sboto-Frankenstein, U., McIver, T., Gervai, P., Wacnik, P., Berrington, N., & Tomanek, B. (2013). Default Mode Network Functional Connectivity Altered in Failed Back Surgery Syndrome. *The Journal of Pain, 14*(5), 483-491. doi:<https://doi.org/10.1016/j.jpain.2012.12.018>

Lee, Y. C., Fine, A., Protsenko, E., Massarotti, E., Edwards, R. R., Mawla, I., . . . Loggia, M. L. (2019). Brain Correlates of Continuous Pain in Rheumatoid Arthritis as Measured by Pulsed Arterial Spin Labeling. *Arthritis Care Res (Hoboken), 71*(2), 308-318. doi:10.1002/acr.23601

Letzen, J. E., Remeniuk, B., Smith, M. T., Irwin, M. R., Finan, P. H., & Seminowicz, D. A. (2020). Individual differences in pain sensitivity are associated with cognitive network functional connectivity following one night of experimental sleep disruption. *Hum Brain Mapp, 41*(3), 581-593. doi:10.1002/hbm.24824

Lim, M., Jassar, H., Kim, D. J., Nascimento, T. D., & DaSilva, A. F. (2021). Differential alteration of fMRI signal variability in the ascending trigeminal somatosensory and pain modulatory pathways in migraine. *The Journal of Headache and Pain, 22*(1), 4. doi:10.1186/s10194-020-01210-6

Lim, M., Nascimento, T. D., Kim, D. J., Ellingrod, V. L., & DaSilva, A. F. (2021). Aberrant Brain Signal Variability and COMT Genotype in Chronic TMD Patients. *J Dent Res, 100*(7), 714-722. doi:10.1177/0022034521994089

López-Solà, M., Geuter, S., Koban, L., Coan, J. A., & Wager, T. D. (2019). Brain mechanisms of social touch-induced analgesia in females. *Pain, 160*(9), 2072-2085. doi:10.1097/j.pain.0000000000001599

Lui, F., Colloca, L., Duzzi, D., Anchisi, D., Benedetti, F., & Porro, C. A. (2010). Neural bases of conditioned placebo analgesia. *Pain, 151*(3), 816-824. doi:10.1016/j.pain.2010.09.021

Ma, X., Li, S., Tian, J., Jiang, G., Wen, H., Wang, T., . . . Xu, Y. (2015). Altered brain spontaneous activity and connectivity network in irritable bowel syndrome patients: A resting-state fMRI study. *Clin Neurophysiol, 126*(6), 1190-1197. doi:10.1016/j.clinph.2014.10.004

Maihöfner, C., Seifert, F., & Decol, R. (2011). Activation of central sympathetic networks during innocuous and noxious somatosensory stimulation. *Neuroimage, 55*(1), 216-224. doi:10.1016/j.neuroimage.2010.11.061

Mainero, C., Boshyan, J., & Hadjikhani, N. (2011). Altered functional magnetic resonance imaging resting-state connectivity in periaqueductal gray networks in migraine. *Ann Neurol, 70*(5), 838-845. doi:10.1002/ana.22537

Makary, M. M., Lee, J., Lee, E., Eun, S., Kim, J., Jahng, G. H., . . . Park, K. (2018). Phantom Acupuncture Induces Placebo Credibility and Vicarious Sensations: A Parallel fMRI Study of Low Back Pain Patients. *Sci Rep, 8*(1), 930. doi:10.1038/s41598-017-18870-1

Mao, C. P., Chen, F. R., Huo, J. H., Zhang, L., Zhang, G. R., Zhang, B., & Zhou, X. Q. (2020). Altered resting-state functional connectivity and effective connectivity of the habenula in irritable bowel syndrome: A cross-sectional and machine learning study. *Hum Brain Mapp, 41*(13), 3655-3666. doi:10.1002/hbm.25038

Mathur, V. A., Moayedi, M., Keaser, M. L., Khan, S. A., Hubbard, C. S., Goyal, M., & Seminowicz, D. A. (2016). High Frequency Migraine Is Associated with Lower Acute Pain Sensitivity and Abnormal Insula Activity Related to Migraine Pain Intensity, Attack Frequency, and Pain Catastrophizing. *Front Hum Neurosci, 10*, 489. doi:10.3389/fnhum.2016.00489

Matsuo, Y., Kurata, J., Sekiguchi, M., Yoshida, K., Nikaido, T., & Konno, S. I. (2017). Attenuation of cortical activity triggering descending pain inhibition in chronic low back pain patients: a functional magnetic resonance imaging study. *J Anesth, 31*(4), 523-530. doi:10.1007/s00540-017-2343-1

Meylakh, N., Crawford, L. S., Mills, E. P., Macefield, V. G., Vickers, E. R., Macey, P. M., . . . Henderson, L. A. (2024). Altered Corticobrainstem Connectivity during Spontaneous Fluctuations in Pain Intensity in Painful Trigeminal Neuropathy. *eneuro, 11*(7). doi:10.1523/ENEURO.0522-23.2024

Mohr, C., Leyendecker, S., Petersen, D., & Helmchen, C. (2012). Effects of perceived and exerted pain control on neural activity during pain relief in experimental heat hyperalgesia: a fMRI study. *Eur J Pain, 16*(4), 496-508. doi:10.1016/j.ejpain.2011.07.010

Moont, R., Crispel, Y., Lev, R., Pud, D., & Yarnitsky, D. (2012). Temporal changes in cortical activation during distraction from pain: a comparative LORETA study with conditioned pain modulation. *Brain Res, 1435*, 105-117. doi:10.1016/j.brainres.2011.11.056

Mosch, B., Hagena, V., Herpertz, S., Ruttorf, M., & Diers, M. (2023). Neural correlates of control over pain in fibromyalgia patients. *Neuroimage Clin, 37*, 103355. doi:10.1016/j.nicl.2023.103355

Moulton, E. A., Pendse, G., Becerra, L. R., & Borsook, D. (2012). BOLD responses in somatosensory cortices better reflect heat sensation than pain. *J Neurosci, 32*(17), 6024-6031. doi:10.1523/jneurosci.0006-12.2012

Moulton, E. A., Pendse, G., Morris, S., Strassman, A., Aiello-Lammens, M., Becerra, L., & Borsook, D. (2007). Capsaicin-induced thermal hyperalgesia and sensitization in the human trigeminal nociceptive pathway: an fMRI study. *Neuroimage, 35*(4), 1586-1600. doi:10.1016/j.neuroimage.2007.02.001

Mungoven, T. J., Marciszewski, K. K., Macefield, V. G., Macey, P. M., Henderson, L. A., & Meylakh, N. (2022). Alterations in pain processing circuitries in episodic migraine. *J Headache Pain, 23*(1), 9. doi:10.1186/s10194-021-01381-w

Naegel, S., Biermann, J., Theysohn, N., Kleinschnitz, C., Diener, H. C., Katsarava, Z., . . . Holle, D. (2018). Polarity-specific modulation of pain processing by transcranial direct current stimulation - a blinded longitudinal fMRI study. *J Headache Pain, 19*(1), 99. doi:10.1186/s10194-018-0924-5

Nahman-Averbuch, H., Martucci, K. T., Granovsky, Y., Weissman-Fogel, I., Yarnitsky, D., & Coghill, R. C. (2014). Distinct brain mechanisms support spatial vs temporal filtering of nociceptive information. *Pain, 155*(12), 2491-2501. doi:10.1016/j.pain.2014.07.008

Oliva, V., Gregory, R., Davies, W. E., Harrison, L., Moran, R., Pickering, A. E., & Brooks, J. C. W. (2021). Parallel cortical-brainstem pathways to attentional analgesia. *Neuroimage, 226*, 117548. doi:10.1016/j.neuroimage.2020.117548

Oshiro, Y., Quevedo, A. S., McHaffie, J. G., Kraft, R. A., & Coghill, R. C. (2007). Brain mechanisms supporting spatial discrimination of pain. *J Neurosci, 27*(13), 3388-3394. doi:10.1523/jneurosci.5128-06.2007

Oshiro, Y., Quevedo, A. S., McHaffie, J. G., Kraft, R. A., & Coghill, R. C. (2009). Brain mechanisms supporting discrimination of sensory features of pain: a new model. *J Neurosci, 29*(47), 14924-14931. doi:10.1523/jneurosci.5538-08.2009

Pando-Naude, V., Barrios, F. A., Alcauter, S., Pasaye, E. H., Vase, L., Brattico, E., . . . Garza-Villarreal, E. A. (2019). Functional connectivity of music-induced analgesia in fibromyalgia. *Sci Rep, 9*(1), 15486. doi:10.1038/s41598-019-51990-4

Peng, W., Zhan, Y., Jin, R., Lou, W., & Li, X. (2023). Aftereffects of alpha transcranial alternating current stimulation over the primary sensorimotor cortex on cortical processing of pain. *Pain, 164*(6), 1280-1290. doi:10.1097/j.pain.0000000000002814

Raij, T. T., Numminen, J., Närvänen, S., Hiltunen, J., & Hari, R. (2009). Strength of prefrontal activation predicts intensity of suggestion-induced pain. *Hum Brain Mapp, 30*(9), 2890-2897. doi:10.1002/hbm.20716

Roy, A., Wang, W. E., Ho, R. L. M., Ribeiro-Dasilva, M. C., Fillingim, R. B., & Coombes, S. A. (2018). Functional brain activity during motor control and pain processing in chronic jaw pain. *Pain, 159*(12), 2547-2564. doi:10.1097/j.pain.0000000000001366

Rütgen, M., Seidel, E. M., Silani, G., Riečanský, I., Hummer, A., Windischberger, C., . . . Lamm, C. (2015). Placebo analgesia and its opioidergic regulation suggest that empathy for pain is grounded in self pain. *Proc Natl Acad Sci U S A, 112*(41), E5638-5646. doi:10.1073/pnas.1511269112

Sandström, A., Ellerbrock, I., Jensen, K. B., Martinsen, S., Altawil, R., Hakeberg, P., . . . Kosek, E. (2019). Altered cerebral pain processing of noxious stimuli from inflamed joints in rheumatoid arthritis: An event-related fMRI study. *Brain Behav Immun, 81*, 272-279. doi:10.1016/j.bbi.2019.06.024

Sandström, A., Ellerbrock, I., Tour, J., Kadetoff, D., Jensen, K., & Kosek, E. (2023). Dysfunctional Activation of the Dorsolateral Prefrontal Cortex During Pain Anticipation Is Associated With Altered Subsequent Pain Experience in Fibromyalgia Patients. *J Pain, 24*(9), 1731-1743. doi:10.1016/j.jpain.2023.05.006

Sandström, A., Ellerbrock, I., Tour, J., Kadetoff, D., Jensen, K. B., & Kosek, E. (2020). Neural correlates of conditioned pain responses in fibromyalgia subjects indicate preferential formation of new pain associations rather than extinction of irrelevant ones. *Pain, 161*(9), 2079-2088. doi:10.1097/j.pain.0000000000001907

Sankarasubramanian, V., Cunningham, D. A., Potter-Baker, K. A., Beall, E. B., Roelle, S. M., Varnerin, N. M., . . . Plow, E. B. (2017). Transcranial Direct Current Stimulation Targeting Primary Motor Versus Dorsolateral Prefrontal Cortices: Proof-of-Concept Study Investigating Functional Connectivity of Thalamocortical Networks Specific to Sensory-Affective Information Processing. *Brain Connect, 7*(3), 182-196. doi:10.1089/brain.2016.0440

Schenk, L. A., & Colloca, L. (2020). The neural processes of acquiring placebo effects through observation. *Neuroimage, 209*, 116510. doi:10.1016/j.neuroimage.2019.116510

Schmidt-Wilcke, T., Ichesco, E., Hampson, J. P., Kairys, A., Peltier, S., Harte, S., . . . Harris, R. E. (2014). Resting state connectivity correlates with drug and placebo response in fibromyalgia patients. *Neuroimage Clin, 6*, 252-261. doi:10.1016/j.nicl.2014.09.007

Schrepf, A., Harper, D. E., Harte, S. E., Wang, H., Ichesco, E., Hampson, J. P., . . . Harris, R. E. (2016). Endogenous opioidergic dysregulation of pain in fibromyalgia: a PET and fMRI study. *Pain, 157*(10), 2217-2225. doi:10.1097/j.pain.0000000000000633

Schwedt, T. J., Chong, C. D., Chiang, C. C., Baxter, L., Schlaggar, B. L., & Dodick, D. W. (2014). Enhanced pain-induced activity of pain-processing regions in a case-control study of episodic migraine. *Cephalalgia, 34*(12), 947-958. doi:10.1177/0333102414526069

Seifert, F., Schuberth, N., De Col, R., Peltz, E., Nickel, F. T., & Maihöfner, C. (2013). Brain activity during sympathetic response in anticipation and experience of pain. *Hum Brain Mapp, 34*(8), 1768-1782. doi:10.1002/hbm.22035

Seminowicz, D. A., & Davis, K. D. (2006). Cortical responses to pain in healthy individuals depends on pain catastrophizing. *Pain, 120*(3), 297-306. doi:10.1016/j.pain.2005.11.008

Sevel, L. S., Craggs, J. G., Price, D. D., Staud, R., & Robinson, M. E. (2015). Placebo analgesia enhances descending pain-related effective connectivity: a dynamic causal modeling study of endogenous pain modulation. *J Pain, 16*(8), 760-768. doi:10.1016/j.jpain.2015.05.001

Sevel, L. S., O'Shea, A. M., Letzen, J. E., Craggs, J. G., Price, D. D., & Robinson, M. E. (2015). Effective connectivity predicts future placebo analgesic response: A dynamic causal modeling study of pain processing in healthy controls. *Neuroimage, 110*, 87-94. doi:10.1016/j.neuroimage.2015.01.056

Shi, Y., Cui, S., Zeng, Y., Huang, S., Cai, G., Yang, J., & Wu, W. (2021). Brain Network to Placebo and Nocebo Responses in Acute Experimental Lower Back Pain: A Multivariate Granger Causality Analysis of fMRI Data. *Front Behav Neurosci, 15*, 696577. doi:10.3389/fnbeh.2021.696577

Shi, Y., Huang, S., Zhan, H., Wang, Y., Zeng, Y., Cai, G., . . . Wu, W. (2020). Personality Differences of Brain Networks in Placebo Analgesia and Nocebo Hyperalgesia: A Psychophysiological Interaction (PPI) Approach in fMRI. *Neural Plast, 2020*, 8820443. doi:10.1155/2020/8820443

Shi, Y., Liu, Z., Zhang, S., Li, Q., Guo, S., Yang, J., & Wu, W. (2015). Brain Network Response to Acupuncture Stimuli in Experimental Acute Low Back Pain: An fMRI Study. *Evid Based Complement Alternat Med, 2015*, 210120. doi:10.1155/2015/210120

Shukla, S., Torossian, A., Duann, J. R., & Leung, A. (2011). The analgesic effect of electroacupuncture on acute thermal pain perception--a central neural correlate study with fMRI. *Mol Pain, 7*, 45. doi:10.1186/1744-8069-7-45

Silvestrini, N., & Corradi-Dell'Acqua, C. (2023). Distraction and cognitive control independently impact parietal and prefrontal response to pain. *Soc Cogn Affect Neurosci, 18*(1). doi:10.1093/scan/nsad018

Song, G. H., Venkatraman, V., Ho, K. Y., Chee, M. W., Yeoh, K. G., & Wilder-Smith, C. H. (2006). Cortical effects of anticipation and endogenous modulation of visceral pain assessed by functional brain MRI in irritable bowel syndrome patients and healthy controls. *Pain, 126*(1-3), 79-90. doi:10.1016/j.pain.2006.06.017

Strigo, I. A., Simmons, A. N., Matthews, S. C., Craig, A. D., & Paulus, M. P. (2008). Association of major depressive disorder with altered functional brain response during anticipation and processing of heat pain. *Arch Gen Psychiatry, 65*(11), 1275-1284. doi:10.1001/archpsyc.65.11.1275

Su, Q., Qin, W., Yang, Q. Q., Yu, C. S., Qian, T. Y., Mouraux, A., . . . Liang, M. (2019). Brain regions preferentially responding to transient and iso-intense painful or tactile stimuli. *Neuroimage, 192*, 52-65. doi:10.1016/j.neuroimage.2019.01.039

Szabo, E., Chang, Y. C., Shulman, J., Sieberg, C. B., Sethna, N. F., Borsook, D., . . . Lebel, A. A. (2022). Alterations in the structure and function of the brain in adolescents with new daily persistent headache: A pilot MRI study. *Headache, 62*(7), 858-869. doi:10.1111/head.14360

Szabo, E., Timmers, I., Borsook, D., Simons, L. E., & Sieberg, C. B. (2022). Altered anterior insula functional connectivity in adolescent and young women with endometriosis-associated pain: Pilot resting-state fMRI study. *Eur J Paediatr Neurol, 41*, 80-90. doi:10.1016/j.ejpn.2022.10.004

Tang, Y., Ren, C., Wang, M., Dai, G., Xiao, Y., Wang, S., . . . Chen, G. (2021). Altered gray matter volume and functional connectivity in patients with herpes zoster and postherpetic neuralgia. *Brain Res, 1769*, 147608. doi:10.1016/j.brainres.2021.147608

Taylor, J. J., Borckardt, J. J., Canterberry, M., Li, X., Hanlon, C. A., Brown, T. R., & George, M. S. (2013). Naloxone-reversible modulation of pain circuitry by left prefrontal rTMS. *Neuropsychopharmacology, 38*(7), 1189-1197. doi:10.1038/npp.2013.13

Theysohn, N., Schmid, J., Icenhour, A., Mewes, C., Forsting, M., Gizewski, E. R., . . . Benson, S. (2014). Are there sex differences in placebo analgesia during visceral pain processing? A fMRI study in healthy subjects. *Neurogastroenterol Motil, 26*(12), 1743-1753. doi:10.1111/nmo.12454

Tong, H., Maloney, T. C., Payne, M. F., Suñol, M., Dudley, J. A., King, C. D., . . . López-Solà, M. (2023). Augmented pain-evoked primary sensorimotor cortex activation in adolescent girls with juvenile fibromyalgia. *Pain, 164*(10), 2316-2326. doi:10.1097/j.pain.0000000000002933

Tsai, Y. H., Yuan, R., Patel, D., Chandrasekaran, S., Weng, H. H., Yang, J. T., . . . Biswal, B. B. (2018). Altered structure and functional connection in patients with classical trigeminal neuralgia. *Hum Brain Mapp, 39*(2), 609-621. doi:10.1002/hbm.23696

Tseng, M. T., Chiang, M. C., Yazhuo, K., Chao, C. C., Tseng, W. I., & Hsieh, S. T. (2013). Effect of aging on the cerebral processing of thermal pain in the human brain. *Pain, 154*(10), 2120-2129. doi:10.1016/j.pain.2013.06.041

Tu, Y., Wilson, G., Camprodon, J., Dougherty, D. D., Vangel, M., Benedetti, F., . . . Kong, J. (2021). Manipulating placebo analgesia and nocebo hyperalgesia by changing brain excitability. *Proc Natl Acad Sci U S A, 118*(19). doi:10.1073/pnas.2101273118

Uematsu, H., Shibata, M., Miyauchi, S., & Mashimo, T. (2011). Brain imaging of mechanically induced muscle versus cutaneous pain. *Neurosci Res, 70*(1), 78-84. doi:10.1016/j.neures.2011.01.015

Valet, M., Sprenger, T., Boecker, H., Willoch, F., Rummeny, E., Conrad, B., . . . Tolle, T. R. (2004). Distraction modulates connectivity of the cingulo-frontal cortex and the midbrain during pain--an fMRI analysis. *Pain, 109*(3), 399-408. doi:10.1016/j.pain.2004.02.033

van der Meulen, M., Kamping, S., & Anton, F. (2017). The role of cognitive reappraisal in placebo analgesia: an fMRI study. *Soc Cogn Affect Neurosci, 12*(7), 1128-1137. doi:10.1093/scan/nsx033

Vanhaudenhuyse, A., Boly, M., Balteau, E., Schnakers, C., Moonen, G., Luxen, A., . . . Faymonville, M. E. (2009). Pain and non-pain processing during hypnosis: a thulium-YAG event-related fMRI study. *Neuroimage, 47*(3), 1047-1054. doi:10.1016/j.neuroimage.2009.05.031

Veldhuijzen, D. S., Nemenov, M. I., Keaser, M., Zhuo, J., Gullapalli, R. P., & Greenspan, J. D. (2009). Differential brain activation associated with laser-evoked burning and pricking pain: An event-related fMRI study. *Pain, 141*(1-2), 104-113. doi:10.1016/j.pain.2008.10.027

Verriotis, M., Sorger, C., Peters, J., Ayoub, L. J., Seunarine, K. K., Clark, C. A., . . . Moayedi, M. (2022). Amygdalar Functional Connectivity Differences Associated With Reduced Pain Intensity in Pediatric Peripheral Neuropathic Pain. *Front Pain Res (Lausanne), 3*, 918766. doi:10.3389/fpain.2022.918766

Wang, T., Zhan, W., Chen, Q., Chen, N., Zhang, J., Liu, Q., . . . Gong, Q. (2016). Altered resting-state ascending/descending pathways associated with the posterior thalamus in migraine without aura. *Neuroreport, 27*(4), 257-263. doi:10.1097/wnr.0000000000000529

Wang, Y., Cao, D. Y., Remeniuk, B., Krimmel, S., Seminowicz, D. A., & Zhang, M. (2017). Altered brain structure and function associated with sensory and affective components of classic trigeminal neuralgia. *Pain, 158*(8), 1561-1570. doi:10.1097/j.pain.0000000000000951

Watson, A., El-Deredy, W., Iannetti, G. D., Lloyd, D., Tracey, I., Vogt, B. A., . . . Jones, A. K. (2009). Placebo conditioning and placebo analgesia modulate a common brain network during pain anticipation and perception. *Pain, 145*(1-2), 24-30. doi:10.1016/j.pain.2009.04.003

Wiech, K., Kalisch, R., Weiskopf, N., Pleger, B., Stephan, K. E., & Dolan, R. J. (2006). Anterolateral prefrontal cortex mediates the analgesic effect of expected and perceived control over pain. *J Neurosci, 26*(44), 11501-11509. doi:10.1523/jneurosci.2568-06.2006

Wittkamp, C. A., Wolf, M. I., & Rose, M. (2024). The neural dynamics of positive and negative expectations of pain. *Elife, 13*. doi:10.7554/eLife.97793

Wolf, M.-I., Wittkamp, C. A., & Rose, M. (2024). Differential neural activity predicts the long-term stability of the effects of positive and negative expectations on pain. *Scientific Reports, 14*(1), 27874. doi:10.1038/s41598-024-77693-z

Younger, J., Aron, A., Parke, S., Chatterjee, N., & Mackey, S. (2010). Viewing pictures of a romantic partner reduces experimental pain: involvement of neural reward systems. *PLoS One, 5*(10), e13309. doi:10.1371/journal.pone.0013309

Youssef, A. M., Gustin, S. M., Nash, P. G., Reeves, J. M., Petersen, E. T., Peck, C. C., . . . Henderson, L. A. (2014). Differential brain activity in subjects with painful trigeminal neuropathy and painful temporomandibular disorder. *Pain, 155*(3), 467-475. doi:10.1016/j.pain.2013.11.008

Youssef, A. M., Macefield, V. G., & Henderson, L. A. (2016). Cortical influences on brainstem circuitry responsible for conditioned pain modulation in humans. *Hum Brain Mapp, 37*(7), 2630-2644. doi:10.1002/hbm.23199

Zambreanu, L., Wise, R. G., Brooks, J. C. W., Iannetti, G. D., & Tracey, I. (2005). A role for the brainstem in central sensitisation in humans. Evidence from functional magnetic resonance imaging. *Pain, 114*(3), 397-407. doi:10.1016/j.pain.2005.01.005

Zeidan, F., Emerson, N. M., Farris, S. R., Ray, J. N., Jung, Y., McHaffie, J. G., & Coghill, R. C. (2015). Mindfulness Meditation-Based Pain Relief Employs Different Neural Mechanisms Than Placebo and Sham Mindfulness Meditation-Induced Analgesia. *J Neurosci, 35*(46), 15307-15325. doi:10.1523/jneurosci.2542-15.2015

Zhang, C., Zhang, Z., Li, Y., Yin, Y., Feng, C., Zhan, W., . . . Wang, C. (2024). Alterations in functional connectivity in patients with non-specific chronic low back pain after motor control exercise: a randomized trial. *Eur J Phys Rehabil Med, 60*(2), 319-330. doi:10.23736/s1973-9087.24.08087-0

Zhang, M., Yang, Z., Zhong, J., Zhang, Y., Lin, X., Cai, H., & Kong, Y. (2022). Thalamocortical Mechanisms for Nostalgia-Induced Analgesia. *J Neurosci, 42*(14), 2963-2972. doi:10.1523/jneurosci.2123-21.2022

Zhang, S., Li, T., Kobinata, H., Ikeda, E., Ota, T., & Kurata, J. (2018). Attenuation of offset analgesia is associated with suppression of descending pain modulatory and reward systems in patients with chronic pain. *Mol Pain, 14*, 1744806918767512. doi:10.1177/1744806918767512

Zhang, Y., Mao, Z., Pan, L., Ling, Z., Liu, X., Zhang, J., & Yu, X. (2018). Dysregulation of Pain- and Emotion-Related Networks in Trigeminal Neuralgia. *Front Hum Neurosci, 12*, 107. doi:10.3389/fnhum.2018.00107

Zhao, M., Chen, L., Cheng, Z., Wang, X., Zhang, S., Li, M., . . . Jia, X. (2025). Altered brain functional connectivity in patients with tension-type headache. *Headache: The Journal of Head and Face Pain, 65*(2), 216-229. doi:<https://doi.org/10.1111/head.14900>

Zhu, Y., Dai, L., Zhao, H., Ji, B., Yu, Y., Dai, H., . . . Ke, J. (2021). Alterations in Effective Connectivity of the Hippocampus in Migraine without Aura. *J Pain Res, 14*, 3333-3343. doi:10.2147/jpr.S327945
